# Development and Biological Evaluation of FL18-Based Novel Antimicrobial Peptide Constructs Against Bacterial Pathogens

**DOI:** 10.1021/acsomega.6c04833

**Published:** 2026-08-13

**Authors:** Neslihan Zencirci, Büşra Kılıç, Öznur Akbal Vural, Gözde Koşarsoy Ağçeli, Cagatay Karaaslan, Ömür Çelikbıçak

**Affiliations:** † Graduate School of Science and Engineering, Polymer Science and Technology Division, 37515 Hacettepe University, Ankara 06800, Turkey; ‡ Faculty of Science, Department of Biology, Molecular Biology Section, Hacettepe University, Ankara 06800, Turkey; § Advanced Technologies Application and Research Center, Hacettepe University, Ankara 06800, Turkey; ∥ Faculty of Science, Department of Biology, Biotechnology Division, Hacettepe University, Ankara 06800, Turkey; ⊥ Faculty of Science, Department of Chemistry, Physical Chemistry Division, Hacettepe University, Ankara 06800, Turkey

## Abstract

Antimicrobial resistance has become a serious global health challenge, increasing the need for new therapeutic strategies beyond conventional antibiotics. In this study, FL18 and its chimeric derivatives, FL18–TAT8 and FL18–Ahx–TAT8, were designed to combine antimicrobial activity with the cell-associated advantages of a cell-penetrating peptide motif and were synthesized by Fmoc-based solid-phase peptide synthesis. Their molecular masses were confirmed by MALDI-MS analysis. The antibacterial activities of the peptides were evaluated against representative Gram-negative and Gram-positive bacterial strains, including *Escherichia coli*, *Pseudomonas aeruginosa*, *Staphylococcus aureus*, and *Enterococcus faecium*. The tested peptides exhibited minimum inhibitory concentration values in the range of 4–64 μM depending on the peptide sequence and bacterial strain, while TAT8 alone showed no significant antibacterial activity under the tested conditions. Among the constructs, FL18–Ahx–TAT8 displayed the most favorable overall antibacterial profile, particularly against *P. aeruginosa* and *E. faecium*. Biocompatibility studies further showed that FL18 and its chimeric derivatives maintained approximately 75–90% HaCaT cell viability across the tested concentration range and exhibited generally low hemolytic activity. In addition, confocal microscopy and flow cytometry revealed peptide-associated fluorescence in HaCaT cells, with the TAT8-containing chimeras showing a broader and more pronounced fluorescence distribution than FL18 alone. Overall, these findings demonstrate that FL18-based chimerization provides a modular and effective strategy for tuning antibacterial activity, biocompatibility, and peptide–cell interaction behavior, underscoring their promise as building blocks for the design of next-generation antimicrobial peptide platforms.

## Graphical abstract

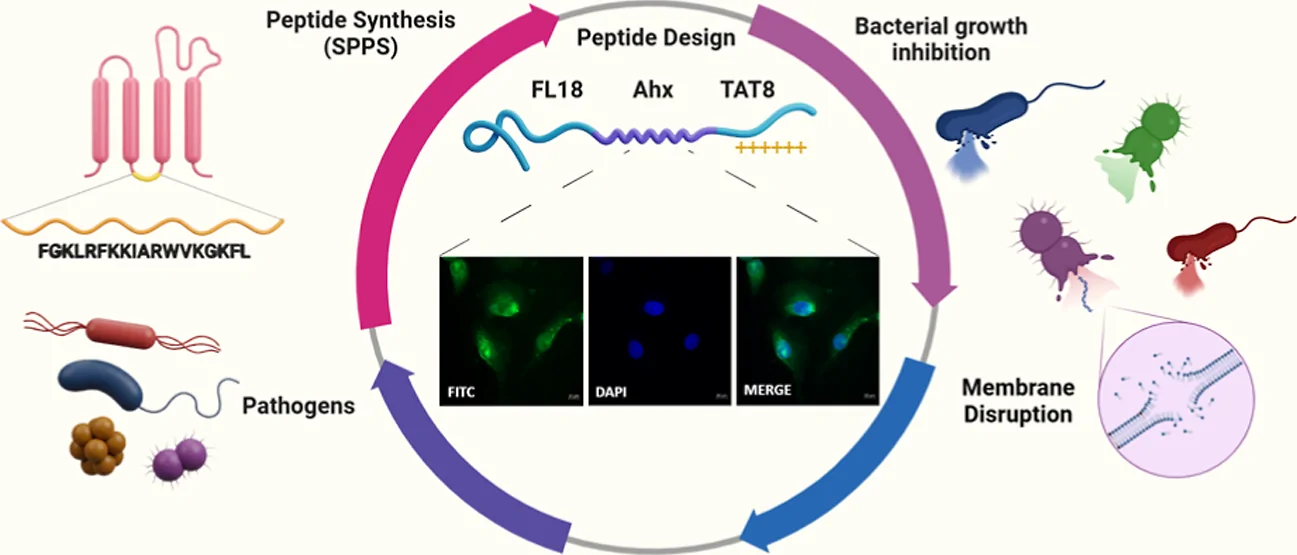

## Introduction

Antimicrobial resistance (AMR) has emerged as one of the foremost public-health challenges of our time, creating an urgent need for new and effective anti-infective strategies. Recent studies have emphasized the growing clinical burden of multidrug-resistant bacterial infections and the declining effectiveness of conventional antibiotics against resistant pathogens.^1,2^ Current projections further suggest that, unless effective interventions are developed, AMR may be responsible for up to 10 million deaths annually by 2050.^3–5^ Particularly troublesome among these are the ESKAPE pathogens, such as *Enterococcus faecium*, *Staphylococcus aureus*, and *Pseudomonas aeruginosa*, which stand out for their striking capacity to withstand or quickly develop resistance against currently used antibiotics.^5,6^ Bacteria can employ multiple resistance mechanisms, including target modification, reduced intracellular accumulation of antibiotics, and enzymatic inactivation of antimicrobial agents, all of which contribute to the diminishing efficacy of current therapies.^7,8^ To address the increasing need for alternative antimicrobial strategies, several classes of nonconventional antimicrobial agents have been investigated, including antimicrobial lipids, cationic polymers, nanoparticles, peptide-based materials, and hybrid nanostructured systems. These platforms can act through diverse mechanisms such as membrane disruption, electrostatic interaction with bacterial envelopes, oxidative stress generation, inhibition of biofilm formation, or controlled delivery of antimicrobial components.^9–11^ Within this landscape, antimicrobial peptides (AMPs) have drawn particular interest as candidate substitutes for standard antibiotics, owing to their wide activity spectrum and their predominantly membrane-targeting modes of action, which may make them less vulnerable to classical resistance mechanisms. AMPs are widely distributed in both prokaryotic and eukaryotic organisms and display activity against bacteria, fungi, viruses, and parasites.^12–16^ Beyond their direct antimicrobial effects, many AMPs are also functionally associated with host defense peptides (HDPs), which may contribute additional biological activities such as immunomodulation, angiogenesis, and wound healing.^17–19^ Structurally, AMPs are generally short oligopeptides composed of approximately 8–50 amino acid residues, with molecular weights commonly ranging from 2 to 10 kDa. They often contain a substantial proportion of hydrophobic amino acids and possess an amphipathic character. In addition, most AMPs carry a net positive charge due to the presence of lysine- and arginine-rich residues, which facilitates electrostatic interaction with negatively charged microbial membranes.^13,16,17^ Based on their structural features, AMPs are typically categorized into several main groups, namely linear α-helical peptides, β-sheet peptides, cyclic peptides, peptides bearing small protein-like motifs, and residue-rich peptides.^20,21^ The growing interest in AMPs has also encouraged the development of synthetic analogues through rational design strategies aimed at optimizing key physicochemical parameters including chain length, overall charge, hydrophobicity, amphipathic balance, and the tendency to adopt defined secondary structures.^22,23^ Such approaches can enhance antimicrobial potency and improve selectivity toward bacterial membranes. Even so, several important limitations still remain, including proteolytic instability, short biological half-life, and undesired cytotoxicity toward mammalian cells.^24^ These challenges have driven the search for multifunctional peptide architectures that can preserve antibacterial activity while improving overall biological performance. At the same time, advances in solid-phase and liquid-phase peptide synthesis have made the practical development of these peptide-based systems considerably more accessible.^22,25,26^ The design of FL18 was guided by database-assisted screening and physicochemical optimization of membrane-active AMP features.^27,28^ Publicly available AMP resources, including the Antimicrobial Peptide Database (APD6), the Database of Antimicrobial Activity and Structure of Peptides (DBAASP),^29^ and the Collection of Antimicrobial Peptides (CAMPR3), were used to evaluate candidate antimicrobial sequence characteristics.^30^ A cecropin–melittin–magainin-inspired sequence framework was selected as the starting point for developing a short 18-residue peptide with favorable cationic character, amphipathic organization, hydrophobic balance, and predicted membrane-interaction potential.^28,31^ In this design, lysine and arginine residues were distributed to strengthen electrostatic interactions with negatively charged bacterial envelopes, whereas hydrophobic and aromatic residues, including phenylalanine, leucine, valine, and tryptophan, were incorporated to support amphipathic organization, membrane association, and potential insertion into lipid-rich environments.^16,19,20,22,23^ The resulting peptide, designated FL18, was therefore designed as a compact hybrid AMP scaffold that integrates key membrane-active physicochemical features of established AMP frameworks into a short and synthetically accessible sequence. In the present study, FL18 and two FL18-based chimeric derivatives were developed to investigate how cell-penetrating peptide (CPP) conjugation and spacer insertion influence antibacterial activity, biocompatibility, and peptide-associated cellular behavior. Two chimeric constructs were generated by incorporating the TAT8 CPP motif either directly or through an aminohexanoic acid (Ahx) spacer into the FL18 framework. Ahx is a flexible aliphatic spacer frequently used in peptide conjugation to provide spatial separation between bioactive domains without introducing an additional charged or highly bulky residue.^32,33^ In FL18–Ahx–TAT8, Ahx was incorporated to reduce potential steric interference between the FL18 antimicrobial scaffold and the TAT8 CPP segment, while maintaining a moderate hydrophobic contribution that may support membrane-associated peptide presentation. Chimeric peptide design provides a modular strategy for combining functionally distinct peptide segments within a single construct. In this context, chimeric peptides are not limited to simple extensions of a single native sequence; rather, they may integrate peptide regions or functional motifs with different biological roles, such as membrane interaction, cell penetration, target recognition, or structural stabilization. Compared with peptides that mimic only one continuous protein region or a single functional segment, chimeric constructs may offer additional advantages by bringing together complementary physicochemical properties within the same molecule. When distinct functional motifs are connected through an appropriate linker or spacer, their spatial separation and conformational presentation can be tuned, which may reduce steric interference, improve accessibility of active regions, and enhance affinity or biological activity in selected systems.^34–36^ Therefore, the design of chimeric peptides can be particularly useful when biological activity depends on the cooperative contribution of more than one functional region. Accordingly, FL18–TAT8 and FL18–Ahx–TAT8 are described in this study as FL18-based chimeric peptide constructs because they combine the antimicrobial FL18 scaffold with the TAT8 cell-penetrating peptide module within a single peptide sequence. In the FL18–Ahx–TAT8 construct, Ahx was incorporated as a spacer to provide additional conformational flexibility and spatial separation between the AMP and CPP segments. TAT8, a truncated derivative of the HIV-1 Tat sequence, was selected as the CPP moiety because of its short arginine-rich structure, strong cationic character, established cell-penetrating properties, and frequent use in peptide delivery and AMP–CPP conjugation strategies.^37–39^ Its compact sequence was also considered advantageous for constructing FL18-based chimeric peptides without excessively increasing the overall peptide length. The resulting constructs, FL18–TAT8 and FL18–Ahx–TAT8, were evaluated against *Escherichia coli*, *P. aeruginosa*, *S. aureus*, and *E. faecium*, as well as for cytocompatibility, hemolytic activity, and peptide-associated cellular fluorescence behavior. Collectively, this study explores whether FL18-based chimerization can serve as a useful modular strategy for tuning antibacterial activity and biological compatibility in AMP design.

## Materials and Methods

### Materials

All reagents used for peptide synthesis were supplied by CEM Corporation (Charlotte, NC, USA). The amino acids employed in the synthesis were obtained with appropriate side chain protecting groups to minimize undesired reactions during stepwise chain elongation. Solvents and other synthesis-grade reagents of the highest commercially available purity were purchased from Sigma-Aldrich (St. Louis, MO, USA).

### Peptide Synthesis and Purification

FL18 was assembled on a Liberty Blue 2.0 automated microwave peptide synthesizer (CEM Corporation, USA) by standard Fmoc-based solid-phase peptide synthesis (SPPS). Briefly, Fmoc-protected amino acids were coupled to Rink amide ProTide resin with *N*,*N*′-diisopropylcarbodiimide (DIC) and ethyl cyano­(hydroxyimino)­acetate (Oxyma) serving as coupling reagents. Fmoc deprotection was carried out in 20% piperidine in dimethylformamide (DMF) with gentle agitation. Chain elongation proceeded through iterative coupling cycles using the same DIC/Oxyma activation strategy, with a 2-fold molar excess of Fmoc-protected amino acids relative to the active sites of the resin. Final cleavage of the peptide from the resin was performed using a trifluoroacetic acid (TFA)/triisopropylsilane (TIPS)/water mixture (95:2.5:2.5, v/v/v) for 2–4 h at room temperature. Peptide molecular masses were confirmed by matrix-assisted laser desorption/ionization time-of-flight mass spectrometry (MALDI-TOF MS; Ultraflex Extreme, Bruker Daltonics Inc., USA) using α-cyano-4-hydroxycinnamic acid as the matrix. FL18 was purified and analyzed by reversed-phase high-performance liquid chromatography (RP-HPLC) on a preparative C18 column. Elution was carried out using a 5–80% ACN (containing 0.1% TFA) gradient over 40 min at a flow rate of 4 mL/min, with detection at 214 nm. The retention time (tR) of each peptide was recorded at the point of maximum peak intensity. Peptide purity exceeded 95%, as confirmed by HPLC. After purification, the peptides were treated with HCl solution to replace trifluoroacetate counterions, then freeze-dried and kept at −20 °C until use. All chimeric peptides were prepared using the same synthetic protocol described for FL18.

### Trypsin-Mediated Proteolytic Stability Assay

The trypsin-mediated proteolytic stability of FL18, TAT8, FL18–TAT8, and FL18–Ahx–TAT8 was evaluated using MALDI-TOF mass spectrometry. Peptide solutions were prepared at a final concentration of 10 μM in ammonium bicarbonate buffer, pH 7.8, and trypsin was added at a peptide: trypsin ratio of 1:40. The mixtures were incubated at 37 °C, and aliquots were collected at 30, 60, 120, 240, and 360 min, as well as after 24 h of incubation. For MALDI-TOF-MS sample preparation, 10 μL of each incubation sample was mixed with the matrix solution at a sample: matrix ratio of 1:10 (v/v). The matrix solution consisted of α-cyano-4-hydroxycinnamic acid prepared at 10 mg/mL in acetonitrile/deionized water (1:1, v/v) containing 0.1% trifluoroacetic acid. The acidic matrix solution was used to stop trypsin activity prior to MALDI-TOF-MS analysis. Immediately after mixing, the samples were spotted onto the MALDI target plate, air-dried, and analyzed after all samples had been prepared. MALDI-TOF-MS measurements were performed using a Bruker Ultraflex Extreme MALDI-TOF mass spectrometer under identical acquisition conditions for all peptide types and time points. Each spectrum was acquired using 1000 laser shots. For each peptide and time point, approximately five independent MALDI spots were prepared and analyzed, and the average signal-to-noise (S/N) ratio of the corresponding parent peptide ion was used to evaluate time-dependent changes in peptide signal intensity under trypsin-containing conditions.

### Circular Dichroism

Circular dichroism (CD) spectra were recorded using a Jasco J-815 spectropolarimeter (Tokyo, Japan) at room temperature under constant nitrogen flow. Spectra were collected across the 190–250 nm range at a scan rate of 50 nm/min using a 1 nm bandwidth. Peptide solutions (50 μM) were prepared from 2 mM stock solutions by dilution either in deionized water or in 30 mM SDS micelles at 25 °C. Each spectrum represents the average of three consecutive scans, after subtraction of the corresponding solvent baseline.

### Zeta Potential

The zeta potentials of aqueous peptide suspensions were measured using a Malvern Zetasizer Nano-ZS (Malvern Instruments Ltd., Malvern, Worcestershire, UK). All measurements were performed at 25 °C using a 633 nm laser. Prior to analysis, samples were diluted to 100 μM with deionized water and sonicated for 2 min to minimize possible aggregation.^40^

### Microorganisms and Cellular Lines

The human epidermal keratinocyte cell line HaCaT (CLS 300493) and the bacterial strains *E. coli* (ATCC 25922), *P. aeruginosa* (ATCC 15442), *E. faecium* (ATCC 19434), and *S. aureus* (ATCC 6538) were obtained from ATCC (Manassas, VA, USA). Dulbecco’s Modified Eagle Medium (DMEM) and MTT reagent were purchased from Sigma-Aldrich (St. Louis, MO, USA). Fetal bovine serum (FBS) and penicillin/streptomycin solution were obtained from Thermo Fisher (Waltham, MA, USA). Nutrient broth and bacteriological agar were purchased from Condalab (Madrid, Spain).

### Antimicrobial Activity Assays

Minimum inhibitory concentration (MIC) values were determined in 96-well plates using the standard broth microdilution method. The antimicrobial activities of the synthesized peptides were evaluated against *E. coli*, *P. aeruginosa*, *S. aureus*, and *E. faecium*. Bacterial inocula were adjusted to a final concentration of 1.5 × 10^6^ CFU/mL. Serial 2-fold peptide dilutions ranging from 4 to 256 μM were prepared in nutrient broth. Plates were incubated at 37 °C for 24 h, after which bacterial growth was assessed by measuring absorbance at 600 nm with a SPECTROstar Nano microplate reader (Offenburg, Germany). All experiments were performed in triplicate. Penicillin/streptomycin was used as the positive drug control.

### Cytotoxicity Assays

HaCaT cells were cultured in DMEM supplemented with 10% FBS, 1% penicillin/streptomycin, and 1% l-glutamine. Cells were seeded into 96-well plates and incubated overnight at 37 °C in a humidified atmosphere containing 5% CO_2_. After incubation, the cells were treated with increasing peptide concentrations (4, 16, 32, 64, and 256 μM) for 24 h, after which cell viability was determined by the MTT [3-(4,5-dimethylthiazol-2-yl)-2,5-diphenyl tetrazolium bromide] assay. Following treatment, the culture supernatants were discarded and the resulting formazan crystals were solubilized in DMSO. Absorbance was measured at 570 nm using an EnSight Multimode Microplate Reader (PerkinElmer, Milan, Italy).^41^

### Hemolytic Assays

Peptide-induced hemolysis was assessed by incubating human red blood cells with peptide solutions at concentrations ranging from 4 to 256 μM. Fresh human red blood cells were obtained from healthy donors and collected in sterile EDTA-coated blood collection tubes. Blood samples were centrifuged at 2000 rpm for 5 min, and the serum fraction was carefully removed. The erythrocyte fraction was washed three times with phosphate-buffered saline (PBS) at 1000*g* for 5 min at 4 °C. After the final wash, the RBC pellet was resuspended in PBS to obtain a 2% (v/v) RBC suspension, which was used for subsequent hemolysis assays. Equal volumes of the peptide dilutions and the 2% RBC suspension were mixed and incubated at 37 °C for 1 h. RBCs suspended in PBS alone represented the negative control, while cells exposed to 1% (v/v) Triton X-100 provided the positive control corresponding to complete lysis. After incubation, the mixtures were centrifuged at 2000–2500 rpm for 5 min, and 200 μL of the resulting supernatant was transferred to a 96-well plate. Each condition was analyzed in triplicate. Hemoglobin release was quantified by measuring the absorbance at 405 nm using a microplate spectrophotometer.^42^

Hemolysis was calculated according to the following equation

$$\%\;hemolysis=\frac{A_{sample}-ANC}{APC-ANC}\times 100$$

### Scanning Electron Microscopy of Bacteria Treated with FL18, TAT8, and FL18 Chimeras

Peptide-induced alterations in bacterial morphology were visualized by scanning electron microscopy (SEM), with samples prepared according to an adapted form of a protocol reported earlier.^43^ Guided by the antimicrobial activity data, peptide doses within the active range (4–32 μM) were chosen for SEM examination. Bacteria were exposed to the peptides at 4 and 32 μM and grown under suitable conditions. Following incubation, the suspensions were pelleted by centrifugation, rinsed twice with phosphate-buffered saline (PBS), and resuspended in PBS. A 5 μL portion of each suspension was deposited onto round glass coverslips. Cells were fixed in 2.5% (v/v) glutaraldehyde prepared in PBS for 45 min at 4 °C, washed twice with PBS, and then dehydrated through an ascending ethanol series (30%, 50%, 70%, 90%, 95%, and 100%), each step lasting 5 min. After dehydration, the specimens were air-dried at room temperature, sputter-coated with gold–palladium, and imaged on a scanning electron microscope (Tescan GAIA3 equipped with Oxford XMax 150 EDS, FIB-SEM system) at the HÜNİTEK Laboratory, Hacettepe University, Ankara.

### Cellular Association and Fluorescence Analysis of FL18, TAT8, and Its Chimeras in HaCaT Cells

For confocal microscopy, HaCaT keratinocytes (50,000 cells) were seeded onto coverslips placed in 6-well plates containing DMEM supplemented with 10% FBS and incubated at 37 °C in 5% CO_2_ for 24 h. After incubation, the cells were washed with PBS and treated with FITC-labeled peptides (16 μM) in serum-free DMEM for 4 h at 37 °C under 5% CO_2_. The cells were then washed again with PBS and fixed with 4% formaldehyde for 10 min at room temperature. After fixation, the cells were washed with PBS, permeabilized with 0.1% Triton X-100 for 10 min, and stained with DAPI (4′,6-diamidino-2-phenylindole, 1 μg/mL) for 15 min at room temperature.^42^ Excess dye was removed by washing with HBSS buffer. Coverslips were mounted onto glass slides and visualized by confocal laser scanning microscopy (Zeiss LSM980). Single-plane confocal images were acquired in this study. DAPI and FITC fluorescence were recorded using excitation/emission wavelengths of 340/488 nm and 498/517 nm, respectively. For flow cytometric analysis, HaCaT cells were seeded in 6-well plates at a density of 1 × 10^5^ cells per well and incubated for 24 h under standard culture conditions. The culture medium was then replaced with medium containing FITC-labeled peptides, while untreated cells were used as the control group. After 1 h of incubation, the cells were detached by trypsinization, centrifuged at 3000 rpm for 3 min, resuspended in PBS, and analyzed by flow cytometry. Data were processed using CytoExpert software (Beckman Coulter, Brea, CA, USA).^43^

### Statistical Analysis

All experiments were performed in three independent experiments (*n* = 3), and data are presented as mean ± SD. Statistical analyses were carried out using GraphPad Prism 11 (GraphPad Software, San Diego, CA, USA). Comparisons among multiple groups were performed using one-way ANOVA followed by Bonferroni’s post hoc test. All tests were two-sided. Statistical significance was defined as follows: ns, not significant (*p* > 0.05); *p < 0.05; **p < 0.01; ***p < 0.001; ****p < 0.0001.

## Results and Discussion

### Peptide Design and Structural Propensity

In the rational design of antimicrobial peptides, cationicity and amphipathicity are widely recognized as key parameters governing peptide–membrane interactions. Initial contact with bacterial membranes is generally driven by electrostatic attraction between positively charged peptide residues and negatively charged membrane surfaces, followed by structural rearrangements that may facilitate membrane association and biological activity.^16,44^ The theoretical molecular weights, net charge values, and hydrophobicity parameters of the peptides investigated in this study are summarized in Table 1, while the overall design strategy and structural characterization are presented in Figure 1. As shown in Figure 1A, FL18–Ahx–TAT8 was constructed as a modular peptide architecture composed of the FL18 antimicrobial segment, the hydrophobic Ahx spacer, and the cell-penetrating TAT8 motif. This design allowed us to examine how CPP conjugation and spacer insertion influence the physicochemical and biological behavior of the parental FL18 scaffold. The surface charge characteristics of the peptides were first evaluated by zeta potential analysis (Figure 1B). FL18 displayed the lowest positive zeta potential, approximately 8 mV, whereas conjugation with TAT8 increased this value to around 13 mV. Notably, FL18–Ahx–TAT8 exhibited the highest zeta potential, reaching approximately 25–30 mV. These findings indicate that structural modification of FL18, particularly through incorporation of the TAT8 segment and the Ahx spacer, substantially enhances the overall cationic character of the peptide constructs. Since electrostatic attraction is one of the major determinants of peptide interaction with bacterial membranes, this increase in positive surface charge may be relevant to the biological differences observed among the tested peptides. The secondary structures of FL18, TAT8, and their chimeric derivatives were then examined by circular dichroism (CD) spectroscopy under both aqueous and membrane-mimetic conditions.^45^ As shown in Figure 1C, FL18 and FL18–TAT8 exhibited characteristic α-helical signatures in the presence of SDS, with double minima near 208 and 222 nm and a positive band around 195 nm. These spectral features are consistent with helical folding under membrane-like conditions. In contrast, FL18–Ahx–TAT8 displayed a markedly weaker helical signature, suggesting that insertion of the Ahx spacer may reduce or destabilize helix formation, possibly by increasing conformational flexibility between the AMP and CPP domains. In deionized water, both FL18 and FL18–TAT8 showed a dominant negative band around 200 nm, indicating a mainly disordered or random coil conformation (Figure 1D). TAT8 alone also displayed a largely disordered spectral profile under both conditions, which is in line with its intrinsically flexible nature.^46^ In combination, these results suggest that FL18 retains the ability to adopt a membrane-associated α-helical conformation, while CPP conjugation and spacer insertion modulate both the charge profile and the structural behavior of the peptide. The difference between the spectra obtained in water and SDS also indicates that the secondary structure of the FL18-based peptides is strongly environment-dependent. In aqueous solution, the peptides predominantly showed disordered conformational features, whereas the anionic SDS micelles promoted α-helical structuring, particularly for FL18 and FL18–TAT8. This behavior is consistent with the conformational plasticity of many cationic amphipathic AMPs, which often remain relatively unstructured in aqueous environments but undergo structure induction upon interaction with negatively charged membrane-mimicking systems.^16,21,25,26,46^ Since SDS micelles provide an anionic interface, they are commonly used as a simplified membrane-mimicking model to evaluate peptide structuring under negatively charged conditions.^25,26,46^ In contrast, neutral or cationic micellar systems would be expected to provide weaker electrostatic driving forces for peptide association, and in cationic environments electrostatic repulsion may further reduce peptide–micelle interaction. Therefore, the extent of helix formation in neutral or cationic micelles may be lower or more dependent on hydrophobic interactions than in SDS. Although such micellar systems were not experimentally examined in the present study, the current CD data support the conclusion that membrane-mimicking anionic environments can promote secondary-structure organization in FL18-based peptide constructs. These changes are likely to contribute to the strain-dependent differences observed later in antibacterial activity and peptide–cell interaction patterns.

**Table 1:** Amino Acid Sequences and Main Physicochemical Properties of FL18, TAT8, and FL18-Based Chimeric Peptides

peptide	sequence	MW (g mol^–1^)	charge	hydrophobicity
FL18	FGKLRFKKIARWVKGKFL	2221.8	+7	–0.206
FL18–TAT8	FGKLRFKKIARWVKGKFLGRKKRRQRR	3288.09	+14	–1.285
FL18–Ahx–TAT8	FGKLRFKKIARWVKGKFL-Ahx-GRKKRRQRR	3401.25	+14	–1.237
TAT8	GRKKRRQRR	1083.29	+7	–3.712

**Figure 1:**
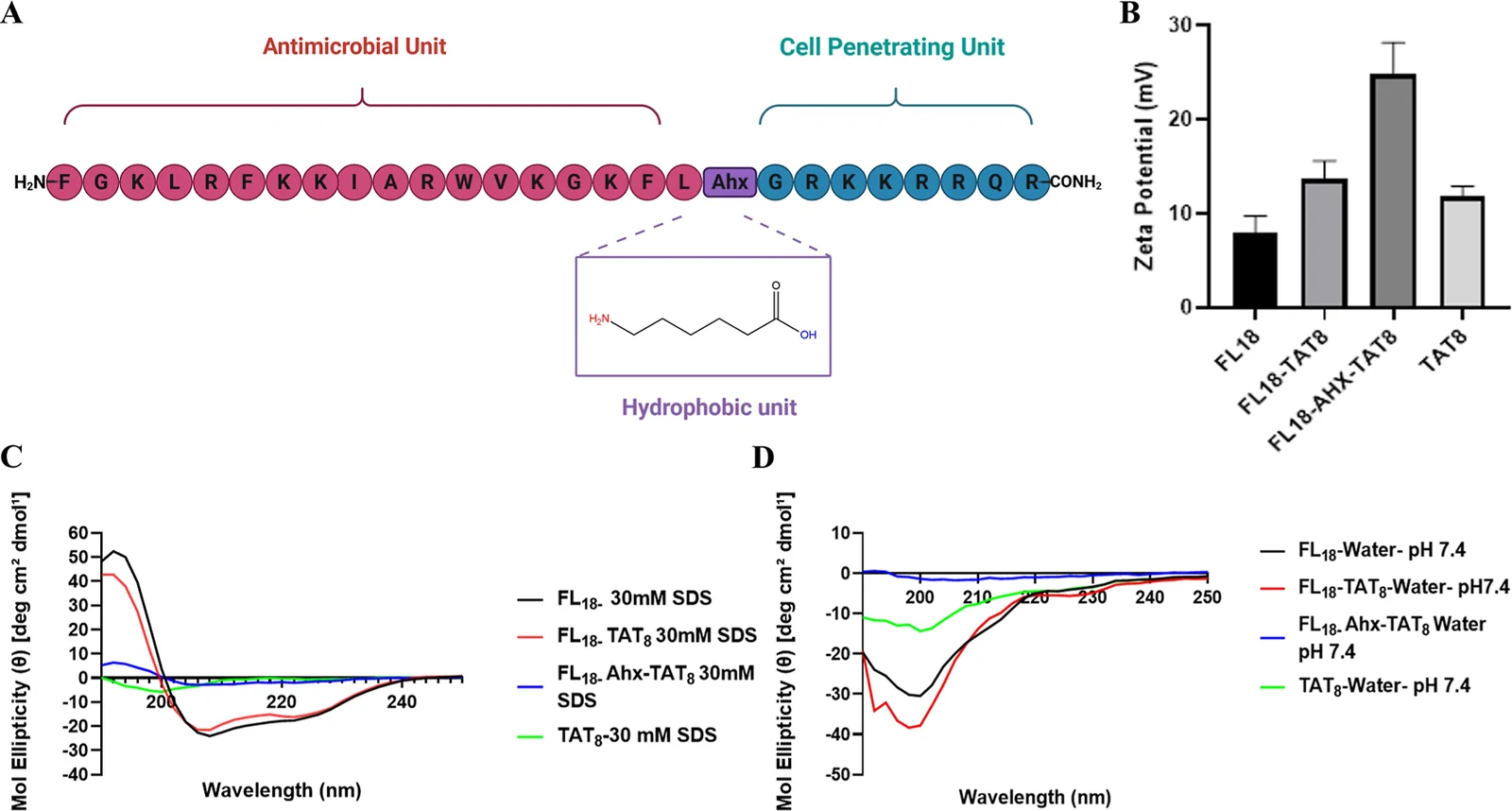
Design and physicochemical characterization of FL18, TAT8, and FL18-based chimeric peptides. (A) Schematic representation of the FL18–Ahx–TAT8 construct. (B) Zeta potential values of FL18, TAT8, FL18–TAT8, and FL18–Ahx–TAT8. (C) Circular dichroism (CD) spectra of the peptides in 30 mM SDS. (D) Circular dichroism (CD) spectra of the peptides in deionized water.

### Trypsin-Mediated Proteolytic Stability

Proteolytic degradation is one of the major limitations affecting the biological applicability of antimicrobial peptides. Therefore, the trypsin-mediated stability of FL18, TAT8, FL18–TAT8, and FL18–Ahx–TAT8 was evaluated using MALDI-TOF mass spectrometry by monitoring time-dependent changes in the parent peptide ion signals. Peptides were incubated under identical trypsin-containing conditions, and MALDI-TOF-MS analyses were performed at 30, 60, 120, 240, and 360 min as well as after 24 h of incubation. For each peptide and time point, multiple MALDI spots were analyzed under the same acquisition parameters, and the average signal-to-noise (S/N) ratios of the corresponding parent ions were used to compare the relative changes in peptide signal intensity over time. Signal-to-noise-based MALDI-MS monitoring has previously been used as a practical approach for evaluating enzymatic activity and peptide/protein signal changes under controlled experimental conditions.^47^ All peptide constructs showed a pronounced decrease in parent ion S/N ratio during the first 60 min of trypsin exposure, indicating that the major reduction in MALDI-detectable peptide signal occurred during the early phase of proteolytic incubation. After this initial decrease, the S/N ratios remained relatively stable from 60 min to 24 h for FL18, TAT8, FL18–TAT8, and FL18–Ahx–TAT8. This pattern suggests an early trypsin-associated loss of parent peptide detectability followed by a comparatively stable residual signal profile over prolonged incubation. The persistence of detectable peptide-related signals after the initial degradation phase may reflect sequence-dependent proteolytic processing, residual parent ion detectability, and/or differences in MALDI ionization behavior among the resulting peptide species. Overall, these results show that all tested peptides are susceptible to trypsin-mediated proteolytic processing, with the most pronounced signal decrease occurring within the first hour of incubation. The use of identical sample preparation, matrix conditions, acquisition parameters, and replicate MALDI spots supports the comparative interpretation of the S/N changes among the peptide groups. Representative MALDI-TOF-MS spectra obtained before and after trypsin incubation are provided in the Supporting Information, Figure S4.

### Antimicrobial Activity Assays

The antibacterial activities of FL18, TAT8, and the FL18-based chimeric peptides were evaluated against representative Gram-negative and Gram-positive bacterial strains, namely *E. coli*, *P. aeruginosa*, *S. aureus*, and *E. faecium*. The corresponding MIC values are presented in Table 2. Penicillin/streptomycin was included as a conventional antibiotic positive control in the antimicrobial assay, and the corresponding control data are now presented in Table 2 to provide a clearer internal reference for antibacterial performance under the same experimental conditions. In addition, representative literature-reported MIC values of standard membrane-active AMPs, including melittin-, LL-37-, magainin-, and cecropin-related peptides, are summarized in Table S1 to provide contextual comparison with the FL18-based constructs. Although direct comparison between independent studies should be interpreted cautiously because MIC values are strongly influenced by bacterial strain, medium composition, inoculum density, peptide purity, counterion form, incubation time, and assay format, the observed antibacterial activity of FL18 and its chimeric derivatives falls within the activity range reported for several short cationic and membrane-active AMP systems. Among the tested sequences, FL18 and its chimeric derivatives showed clear antibacterial activity, whereas TAT8 alone did not exhibit meaningful inhibitory activity under the tested conditions, with MIC values above 256 μM for all strains. This finding indicates that the CPP segment alone does not confer intrinsic antibacterial activity under the tested conditions.^48^ FL18 was active against both Gram-negative and Gram-positive bacteria, showing MIC values of ≤16 μM toward *E. coli* and *P. aeruginosa* and ≤64 μM toward *S. aureus* and *E. faecium*. This pattern suggests that the parental FL18 peptide is more effective against the tested Gram-negative strains than against the Gram-positive strains. After conjugation with TAT8, the antibacterial profile changed in a strain-dependent manner. FL18–TAT8 retained activity against *P. aeruginosa* (≤16 μM) and showed improved activity against *S. aureus* and *E. faecium* (both ≤16 μM), although its activity against *E. coli* decreased relative to FL18 (≤64 μM). This result suggests that CPP fusion does not uniformly enhance antimicrobial performance but rather alters the behavior of the parental scaffold depending on the bacterial target. Among all constructs, FL18–Ahx–TAT8 exhibited the most favorable overall antibacterial profile. In particular, it was most potent against *P. aeruginosa* and *E. faecium*, for which the MIC reached ≤4 μM in both cases. Although its activity against *E. coli* (≤32 μM) and *S. aureus* (≤32 μM) was not uniformly superior to every other construct, the inclusion of the Ahx spacer appears to provide a beneficial structural effect in selected bacterial contexts. In this respect, the spacer may help optimize the balance between charge distribution, conformational flexibility, and functional presentation of the AMP and CPP segments. The hydrophobicity of the spacer is also likely to influence the balance between solubility, conformational flexibility, membrane association, and biocompatibility. A less hydrophobic spacer may improve aqueous compatibility but could reduce hydrophobic interactions that contribute to membrane-associated presentation of the peptide domains. Conversely, a more hydrophobic spacer may enhance membrane affinity but could also increase aggregation tendency, nonspecific membrane disruption, cytotoxicity, or hemolytic activity. Therefore, Ahx was selected as a balanced spacer for this first-generation FL18-based chimeric design, while systematic comparison of spacers with different hydrophobicities will be valuable for future structure–activity optimization. Previous reports have largely emphasized the benefits of AMP–CPP conjugation strategies in Gram-negative systems.^49–51^ In the present study, however, the chimeric peptides also showed meaningful activity against Gram-positive strains, suggesting that the effects of this design strategy may extend beyond a narrower Gram-negative framework. Overall, the antibacterial data indicate that both CPP conjugation and spacer insertion can reshape the biological behavior of the FL18 scaffold, but the extent of improvement depends strongly on the specific peptide–bacterium combination. Within this series, FL18–Ahx–TAT8 emerged as the most promising construct for further evaluation. These strain-dependent MIC differences indicate that the antibacterial performance of FL18-based constructs is governed not only by peptide sequence but also by species-specific bacterial envelope properties and peptide–surface interactions. The strain-dependent antibacterial profiles observed in this study may be associated with differences in bacterial envelope architecture, surface charge distribution, and peptide structural organization.^16,19,20,44^ FL18 showed comparatively stronger activity against the tested Gram-negative strains, which may be related to favorable electrostatic and hydrophobic interactions with the negatively charged outer membrane and lipopolysaccharide-rich surface of Gram-negative bacteria.^16,44^ In contrast, FL18–TAT8 displayed improved activity against the tested Gram-positive strains, suggesting that conjugation with the highly cationic TAT8 motif altered the interaction profile of the parental FL18 scaffold. The thick peptidoglycan layer and anionic teichoic acid components of Gram-positive bacterial envelopes may influence peptide binding, local accumulation, and accessibility to the cytoplasmic membrane.^16,20^ Moreover, the enhanced activity of FL18–Ahx–TAT8 against *E. faecium* may be partly associated with the conformational flexibility provided by the Ahx spacer, which can reduce steric interference between the AMP and CPP domains and support a more favorable spatial presentation of the functional peptide segments.^32,33,52^ Although direct lipid-binding, membrane permeabilization, or pore-formation assays were not performed, these findings suggest that bacterial envelope composition, charge distribution, and peptide conformational organization collectively contribute to the observed strain-dependent antimicrobial behavior.

**Table 2:** MIC Values of FL18, TAT8, FL18-Based Chimeric Peptides, and the Antibiotic Positive Control Against the Tested Bacterial Strains

peptide	E. coli (ATCC 25922) (μM)	P. aeruginosa (ATCC 15442) (μM)	S. aureus (ATCC 6538) (μM)	E. faecium (ATCC 19434) (μM)
FL18	≤16	≤16	≤64	≤64
FL18–TAT8	≤64	≤16	≤16	≤16
FL18–Ahx–TAT8	≤32	≤4	≤32	≤4
TAT8	>256	>256	>256	>256
penicillin/streptomycin	≤4	≤4	≤4	≤4

### Biocompatibility Assays

The biocompatibility of FL18, TAT8, and the FL18-based chimeric peptides was assessed by measuring cytotoxicity toward the human keratinocyte cell line HaCaT and hemolytic activity against RBCs. The corresponding results are shown in Figure 2. The cytotoxicity data revealed a concentration-dependent decrease in HaCaT cell viability for FL18 and its chimeric derivatives. Nevertheless, cell viability remained above approximately 75% across the tested concentration range for all peptide groups, indicating an overall acceptable level of cytocompatibility under the experimental conditions (Figure 2A–D). The antibacterial action of cationic antimicrobial peptides is generally ascribed to their direct engagement with microbial membranes, which compromises membrane integrity. Many cationic peptides also display lower affinity for mammalian membranes, thereby contributing to reduced toxicity. Therefore, the low toxicity observed in the present study may be associated with the selective membrane interactions of these peptides.^53–55^ These findings are particularly important when considered together with the MIC values, as they suggest that antibacterial activity was achieved without a pronounced loss of compatibility toward mammalian cells. The hemolysis results further highlighted notable differences among the tested peptides. Many recently developed antimicrobial peptide candidates have shown that high antimicrobial selectivity can be achieved together with low hemolytic activity, emphasizing the importance of balancing bioactivity and biocompatibility during peptide design.^56^ In this context, rational modulation of physicochemical parameters such as hydrophobicity, cationic charge density, amphipathicity, and residue distribution has emerged as an effective strategy to improve bacterial targeting while reducing toxicity toward erythrocytes and other mammalian cells.^42,57,58^ FL18 and its chimeric derivatives exhibited generally low hemolytic activity throughout the tested concentration range, with HC10 values above 256 μM, indicating limited membrane-disruptive effects toward RBCs (Figure 2E–G). In contrast, TAT8 alone displayed comparatively higher hemolytic activity, reaching about 25% hemolysis at 32 μM and above (Figure 2H). In particular, incorporation of the CPP motif did not lead to a marked increase in erythrocyte membrane disruption, indicating that conjugation enhanced functionality without compromising compatibility.^49^ This observation suggests that although TAT8 alone may show undesirable membrane activity toward mammalian cells, its incorporation into the FL18 scaffold substantially reduces this unfavorable effect. When cytotoxicity, hemolysis, and antibacterial data are considered together, the FL18-based constructs appear to maintain a relatively favorable balance between antimicrobial activity and biological compatibility. In particular, the low hemolytic activity of FL18, FL18–TAT8, and FL18–Ahx–TAT8, together with their retained antibacterial potency, supports the suitability of FL18 as a promising scaffold for further AMP optimization. Importantly, the improved biological profiles of the chimeric constructs did not appear to come at the expense of mammalian cell selectivity, which is a desirable feature for future peptide engineering efforts.

**Figure 2:**
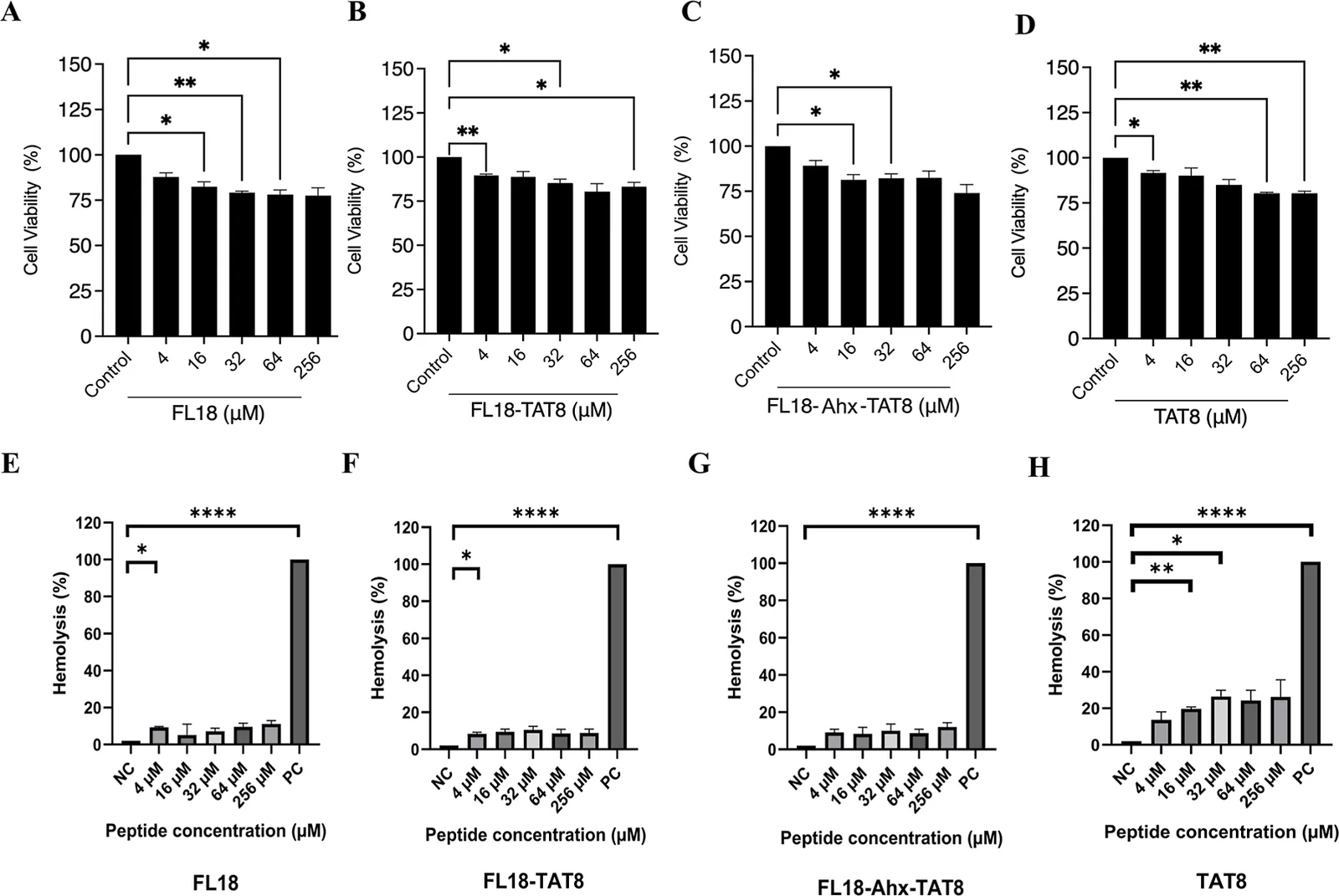
Biocompatibility profile of FL18, TAT8, and FL18-based chimeric peptides. Cytotoxicity in HaCaT cells after 24 h treatment with (A) FL18, (B) FL18–TAT8, (C) TAT8, and (D) FL18–Ahx–TAT8. Hemolytic activity of (E) FL18, (F) FL18–TAT8, (G) FL18–Ahx–TAT8, and (H) TAT8 against human red blood cells. Statistical analysis was performed using one-way ANOVA followed by Bonferroni’s post hoc test. Statistical significance was defined as follows: ns, not significant (*p* > 0.05); *p < 0.05; **p < 0.01; ***p < 0.001; ****p < 0.0001.

### Cellular Association and Fluorescence Distribution in HaCaT Cells

The peptide-associated cellular behavior of FL18, TAT8, FL18–TAT8, and FL18–Ahx–TAT8 in HaCaT cells was further investigated by confocal laser scanning microscopy and flow cytometry using FITC-labeled peptides. Representative confocal images are shown in Figure 3, where peptide-associated fluorescence is shown in green and cell nuclei are counterstained with DAPI in blue. DAPI staining indicated that nuclei largely retained their regular oval morphology in all treatment groups, with no obvious evidence of chromatin condensation or nuclear fragmentation under the tested conditions. This visual observation is consistent with the relatively favorable cytocompatibility results obtained in the viability assays. More importantly, clear differences were observed in the intracellular fluorescence distribution patterns of the peptide groups. FL18 produced a weaker and more spatially restricted green fluorescence signal than the other peptides (Figure 3A–C), suggesting more limited cellular association or a less extensive intracellular fluorescence distribution. In contrast, the TAT8-containing peptides, particularly FL18–TAT8 and FL18–Ahx–TAT8, displayed stronger and more broadly distributed fluorescence throughout the cell body (Figure 3G–L), while TAT8 alone also showed a distinct peptide-associated fluorescence signal (Figure 3D–F). These observations are in line with the expected contribution of the arginine-rich CPP motif to peptide–cell interaction behavior.^17,39^ Among the chimeric constructs, FL18–Ahx–TAT8 showed the most homogeneous and widespread fluorescence pattern. This may indicate that insertion of the Ahx spacer promotes peptide-associated cellular interaction by providing greater structural flexibility and more effective spatial separation between the AMP and CPP segments. By contrast, the parental FL18 peptide, which lacks a CPP domain, showed a more limited fluorescence distribution pattern. Overall, these results suggest that conjugation with TAT8, and to some extent the presence of the Ahx spacer, alters the way FL18 interacts with cells and how peptide-associated fluorescence is distributed within the cellular environment. To complement the imaging data, peptide-associated fluorescence was also quantified by flow cytometry after 1 h incubation at 16 μM (Figure 4). All FITC-labeled peptides produced substantial fluorescence signals in HaCaT cells, confirming effective peptide–cell association under the tested conditions. While only modest differences were observed among FL18, FL18–TAT8, and FL18–Ahx–TAT8 in terms of total fluorescence intensity, the confocal images revealed more pronounced differences in fluorescence distribution. This distinction is important, as it suggests that CPP conjugation did not simply increase overall signal intensity, but rather influenced how the peptide-associated signal was spatially distributed within the cells.^59,60^ This effect was most evident for FL18–Ahx–TAT8, indicating that the combination of an AMP core, a CPP motif, and a flexible spacer may be advantageous for enhancing peptide–cell interaction behavior. At the same time, these findings should be interpreted cautiously as evidence of enhanced cellular association rather than definitive proof of intracellular delivery mechanism or specific subcellular targeting.

**Figure 3:**
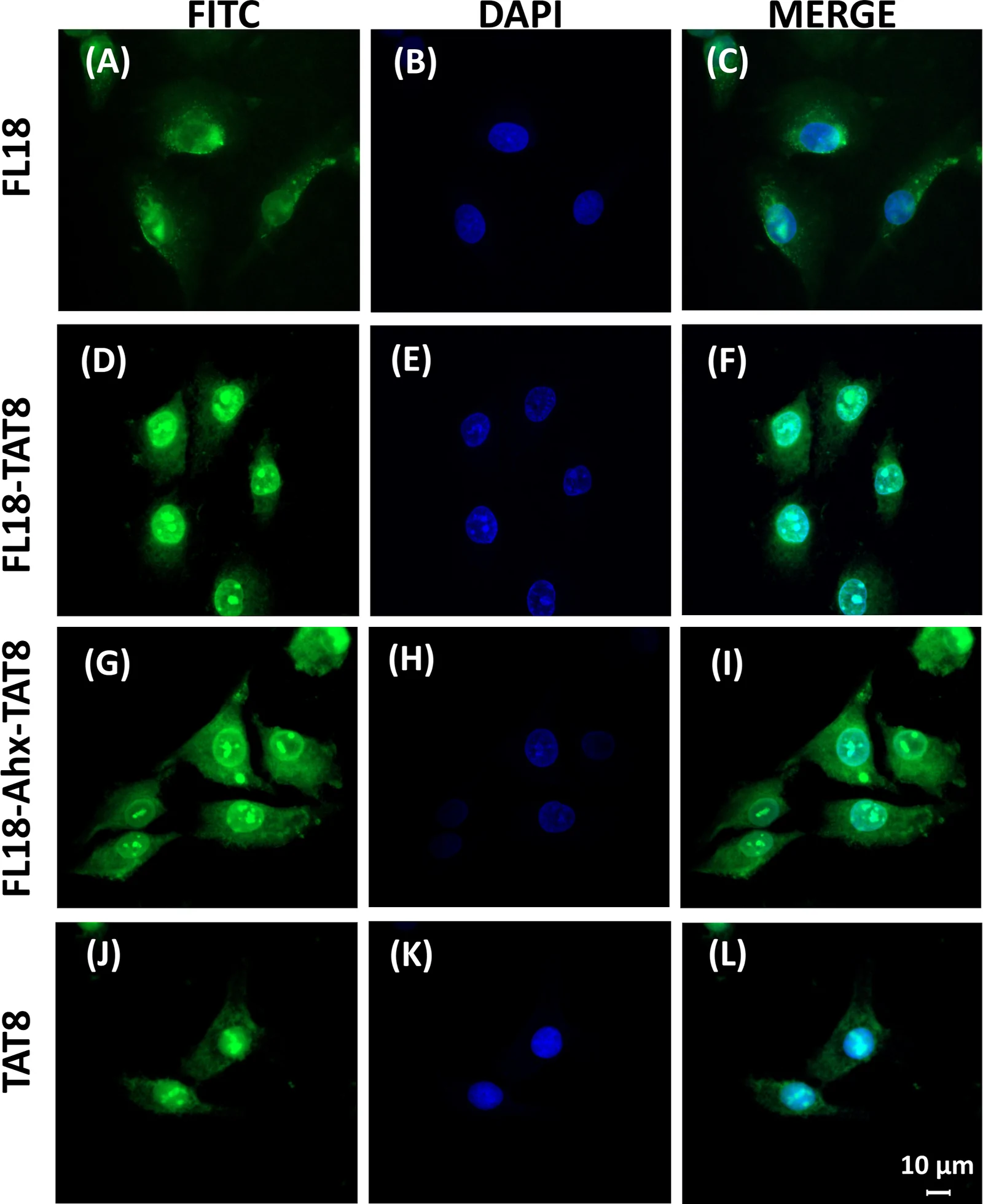
Confocal laser scanning microscopy images of HaCaT cells treated with FITC-labeled peptides (16 μM) for 4 h. Representative images are shown for FL18 (A–C), FL18-TAT8 (D–F), FL18–Ahx-TAT8 (G–I), and TAT8 (J–L). Nuclei were stained with DAPI (blue), and peptide-associated fluorescence is shown in green. Scale bars: 10 μm.

**Figure 4:**
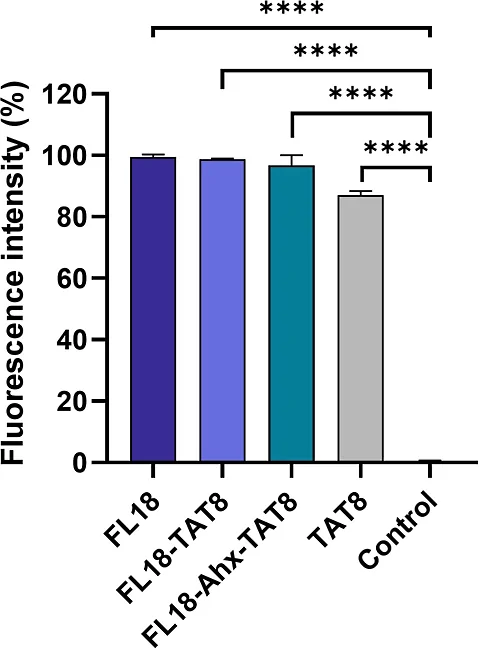
Peptide-associated cellular fluorescence of FITC-labeled FL18, TAT8, and FL18-based chimeric peptides in HaCaT cells, analyzed by flow cytometry after 1 h incubation at 25 °C in PBS (pH 7.4). Statistical analysis was performed using one-way ANOVA followed by Bonferroni’s post hoc test. Statistical significance was defined as follows: ns, not significant (*p* > 0.05); *p < 0.05; **p < 0.01; ***p < 0.001; ****p < 0.0001.

### Morphological Alterations of Bacterial Cell Membranes

To further evaluate the antibacterial effects of the peptides at the cellular level, scanning electron microscopy (SEM) was performed after 24 h treatment of Gram-negative and Gram-positive bacteria with FL18, TAT8, FL18–TAT8, and FL18–Ahx–TAT8 at concentrations of 4 and 32 μM. Representative SEM images are presented in Figures 5 and 6. In the untreated control groups, all bacterial strains retained their characteristic morphology, displaying smooth surfaces and well-defined cellular boundaries. In contrast, peptide-treated groups exhibited different degrees of surface disruption and structural damage depending on peptide composition and concentration. For the Gram-negative strains, peptide treatment caused evident changes in cell surface architecture and overall cellular integrity. FL18 induced visible but moderate morphological perturbations, particularly at 32 μM, where cells showed surface irregularities and partial deformation. TAT8 alone caused relatively limited visible damage, and most cells largely preserved their rod-shaped morphology. By contrast, the chimeric peptides FL18–TAT8 and FL18–Ahx–TAT8 produced more pronounced ultrastructural alterations. Even at 4 μM, these peptides caused detectable surface roughening and localized deformation in some cells. At 32 μM, the damage became much more evident, including distorted bacillary morphology, wrinkled or corrugated cell surfaces, collapse-like appearances, and fragmented or debris-like material surrounding damaged cells (Figure 5). A similar, and in some cases more pronounced, pattern was observed for the Gram-positive strains. Untreated cells maintained their typical coccoid morphology with smooth and continuous surfaces, whereas peptide-treated groups displayed progressive deformation and loss of structural integrity. FL18 caused moderate surface perturbation and distortion of the normal spherical morphology, especially at the higher concentration. TAT8 again showed comparatively weak visible effects.^39,61^ In contrast, FL18–TAT8 and FL18–Ahx–TAT8 caused marked damage, including surface collapse, irregular cell contours, wrinkling, shrinkage, and amorphous debris-like remnants. In several micrographs obtained at 32 μM, the original coccoid morphology was severely disrupted, indicating extensive surface and envelope-associated damage (Figure 6). Overall, the SEM observations consistently showed that the chimeric peptides exerted stronger morphological effects on bacterial cells than FL18 or TAT8 alone. These effects were observed in both Gram-negative and Gram-positive bacteria and became more pronounced at higher concentration. The visible disruption of cell surfaces, deformation of cellular architecture, and loss of structural integrity support the membrane-active antibacterial behavior of the FL18-based chimeric constructs and are in good agreement with the MIC findings. Together with the CD results, the SEM observations support a membrane-associated antibacterial mechanism for the FL18-based chimeric peptides. The α-helical structuring observed in anionic SDS micelles suggests that FL18-containing constructs can undergo conformational organization in negatively charged membrane-mimicking environments, which may facilitate interaction with bacterial envelopes and contribute to local membrane packing disturbance, surface deformation, and loss of structural integrity. However, the present data do not directly demonstrate membrane insertion or pore formation. Therefore, pore formation, carpet-like disruption, or detergent-like destabilization should be regarded as plausible but unconfirmed mechanisms that require further validation using dedicated membrane permeabilization, depolarization, vesicle leakage, or peptide–lipid interaction assays.

**Figure 5:**
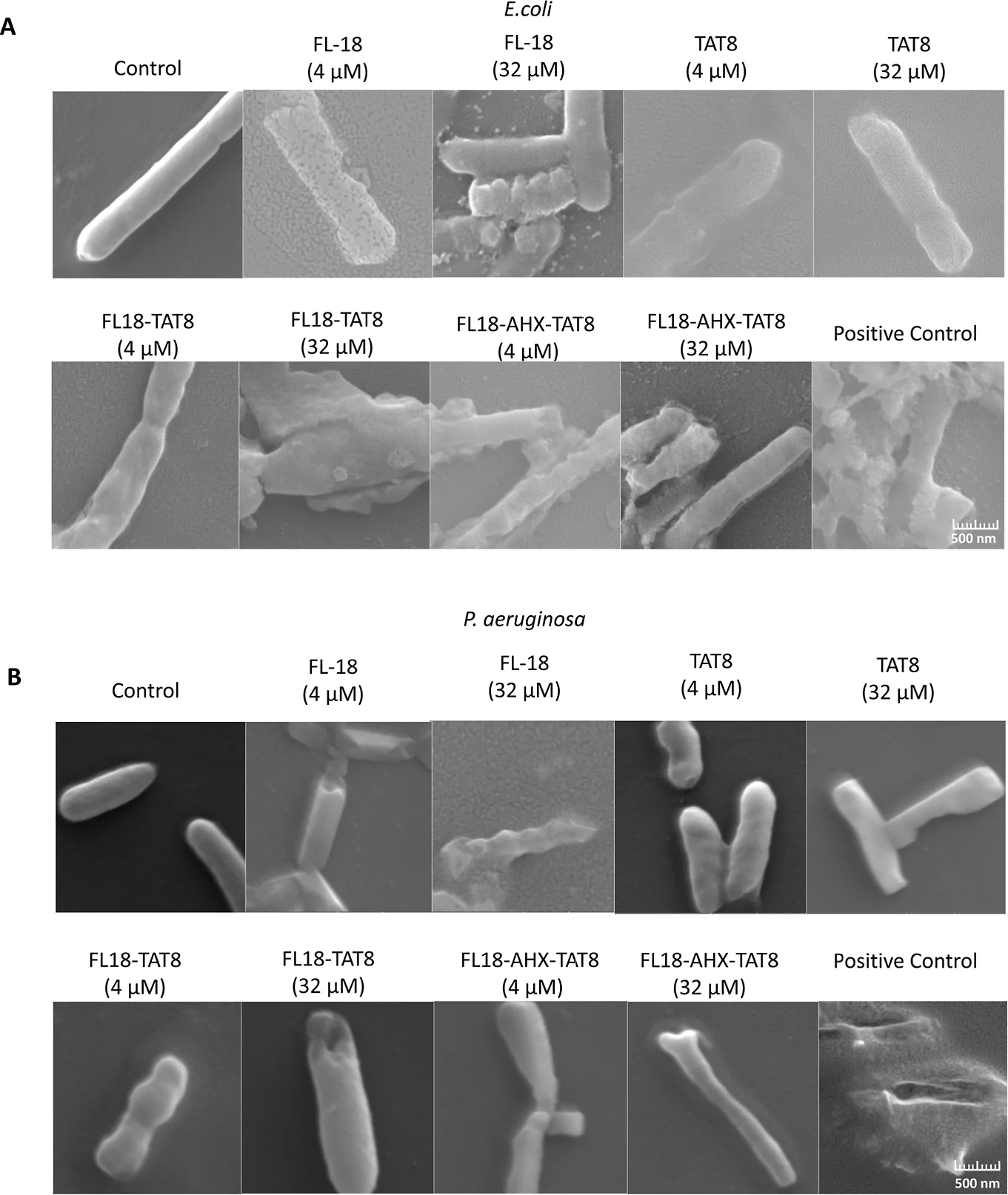
Scanning electron microscopy images of Gram-negative bacteria after 24 h treatment with FL18, TAT8, FL18–TAT8, and FL18–Ahx–TAT8 at 4 or 32 μM. (A) *Escherichia coli* and (B) *Pseudomonas aeruginosa*.

**Figure 6:**
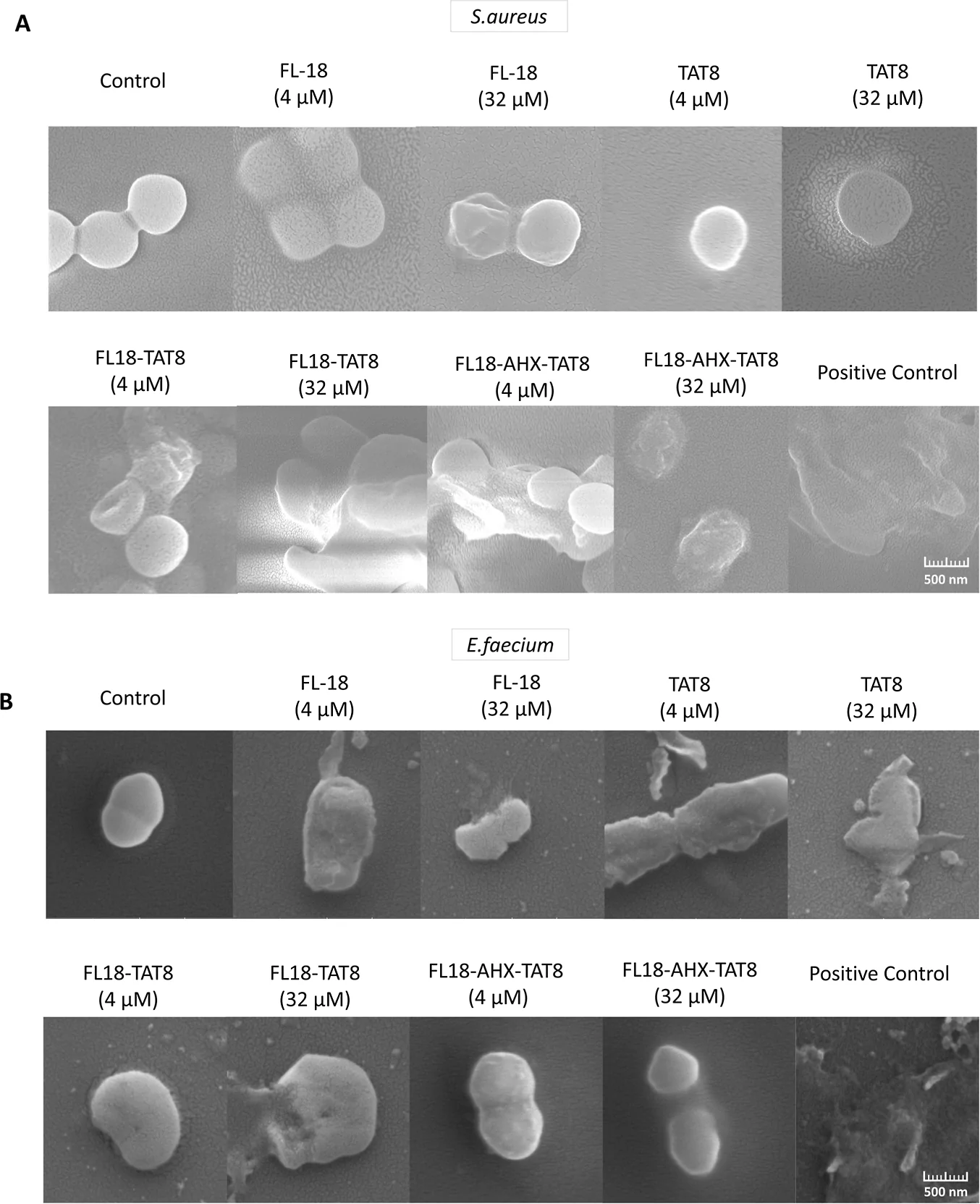
Scanning electron microscopy images of Gram-positive bacteria after 24 h treatment with FL18, TAT8, FL18–TAT8, and FL18–Ahx–TAT8 at 4 or 32 μM. (A) *Staphylococcus aureus* and (B) *Enterococcus faecium*.

## Conclusion

In this study, FL18-based chimeric peptides incorporating the TAT8 cell-penetrating motif, either directly or through an Ahx spacer, were rationally designed and successfully synthesized in order to investigate how structural modification influences antibacterial activity, biocompatibility, and peptide-associated cellular behavior. The findings showed that CPP incorporation did not lead to a uniform improvement across all tested bacterial strains but instead produced sequence- and strain-dependent changes in biological performance. Among the peptide constructs evaluated, FL18–Ahx–TAT8 exhibited the most favorable overall profile, particularly with respect to antibacterial activity against *P. aeruginosa* and *E. faecium*, while maintaining low hemolytic activity and acceptable compatibility with HaCaT cells. In contrast, TAT8 alone showed weak antibacterial activity together with a comparatively higher hemolytic effect, suggesting that its contribution is more beneficial when incorporated into a rationally designed AMP scaffold rather than used as an isolated sequence. Confocal microscopy and flow cytometry further indicated that TAT8-containing chimeras displayed a broader and more pronounced peptide-associated fluorescence pattern in HaCaT cells than FL18 alone, pointing to altered peptide–cell interaction behavior following CPP conjugation. At the same time, these observations should be interpreted carefully as evidence of enhanced cellular association rather than direct proof of a specific uptake mechanism or intracellular targeting pathway. Overall, the present results demonstrate that FL18-based chimerization provides a useful modular approach for tuning antibacterial activity and biological compatibility in antimicrobial peptide design. In particular, the incorporation of a flexible spacer such as Ahx appears to offer an additional level of structural optimization in selected peptide systems. Taken together, these findings support the further development of FL18-derived chimeric peptides as promising scaffolds for next-generation antimicrobial peptide engineering. The present findings obtained with well-characterized ATCC reference strains provide a reproducible experimental basis for further evaluation of FL18-based chimeric peptides against multidrug-resistant clinical isolates. Future studies focusing on resistant clinical strains will be valuable for further defining the antibacterial spectrum and application potential of these peptide constructs.

## Supplementary Material



## Notes

Table S1. Minimum inhibitory concentration (MIC) values of selected reference antimicrobial peptides; MALDI-TOF mass spectra of peptides; preparative RP-HPLC chromatograms; analytical HPLC chromatograms; MALDI-TOF-MS spectra of peptides before and after trypsin incubation; MIC graphs of FL18, FL18–TAT8, FL18–Ahx–TAT8, and TAT8; confocal microscopy images; and flow cytometry histograms (PDF)

## Abbreviations

AMPs: antimicrobial peptides
AMR: antimicrobial resistance
HDPs: host defense peptides
CPP: cell-penetrating peptide
APD6: Antimicrobial Peptide Database
DBAASP: Database of Antimicrobial Activity and Structure of Peptides
CAMPR3: Collection of Antimicrobial Peptides
HIV-1: Human Immunodeficiency Virus type 1
TAT: Trans-Activator of Transcription
SPPS: solid phase peptide synthesis
Fmoc: 9-fluorenylmethoxycarbonyl
DIC: *N*,*N*′-diisopropylcarbodiimide
Oxyma: ethylcyano­(hydroxyimino)­acetate
Ahx: aminohexanoic acid
DMEM: Dulbecco’s modified Eagle medium
FBS: fetal bovine serum
PBS: phosphate buffered saline
DAPI: 4′,6-diamidino-2-phenylindole
MTT: 3-(4,5-dimethylthiazol-2 yl)-2,5-diphenyl tetrazolium
HaCaT: human epidermal keratinocyte cell line
RBCs: red blood cells
MIC: minimum inhibitory concentration
CD: circular dichroism
SEM: scanning electron microscopy
