# Supplementary material for: Development and Biological Evaluation of FL18-Based Novel Antimicrobial Peptide Constructs Against Bacterial Pathogens
Source: ACS Omega. 2026 Aug 13;11(33):49924–37. doi: 10.1021/acsomega.6c04833 (PMC13520428; doi:10.1021/acsomega.6c04833)
Supplement: Supplementary file 1 [file ao6c04833_si_001.pdf]

Supporting Information for

Development and Biological Evaluation of FL18-Based Novel Antimicrobial Peptide Constructs Against Bacterial Pathogens

Neslihan Zencirci[1], Büşra Kılıç[2], Öznur Akbal Vural[3], Gözde Koşarsoy Ağçeli[4], Cagatay Karaaslan[2], Ömür Çelikbıçak[1,5]*

[1]Hacettepe University, Graduate School of Science and Engineering, Department of Polymer Science and Technology Division, Ankara, Turkey.

[2]Hacettepe University, Faculty of Science, Department of Biology, Molecular Biology Section, Ankara, Turkey.

[3]Hacettepe University, Advanced Technologies Application and Research Center, Ankara, Turkey.

[4]Hacettepe University, Faculty of Science, Department of Biology, Biotechnology Division, Ankara, Turkey.

[5]Hacettepe University, Faculty of Science, Department of Chemistry, Physical Chemistry Division, Ankara, Turkey.

*Corresponding Author Email: omurce@hacettepe.edu.tr

Table of Contents

Table S1. Minimum inhibitory concentration (MIC) values of selected reference antimicrobial peptides.. S3

Figure S1 MALDI-TOF Mass spectrum of peptides... S4

Figure S2 RP-HPLC chromatograms of synthesized peptides... S5

Figure S3 Analytical HPLC chromatograms of synthesized peptides...................................... S6

Figure S4 MALDI-TOF Mass spectra of peptides before and after 24 h of trypsin incubation S7

Figure S5 MIC graph of FL18... S8

Figure S6 MIC graph of FL18-TAT8...S9

Figure S7 MIC graph of FL18-Ahx-TAT8...S10

Figure S8 MIC graph of TAT8..S11

Figure S9 Confocal microscopy images...S12

Figure S10 Flow cytometry histograms...S13

References...S14

Table S1. Minimum inhibitory concentration (MIC) values of selected reference antimicrobial peptides (melittin, magainin, and cecropin) against representative Gram-positive and Gram-negative bacterial strains.

Peptide	*E. coli*	*P. aeruginosa*	*S. aureus*	*E. faecium*	References
**Cecropin A (1–8)**	≤64 µM (ATCC 25922)	≤64 µM (ATCC 27853)	>64 µM (ATCC 29213)	Not specified	[1, 2]
**Cecropin A (2-8) + Melittin (6-9)**	≤4 µM (ATCC 25922)	≤8 µM (ATCC 27853)	8 µM (ATCC 25923)	Not specified	[3]
**Magainin 2**	≤40 µM	>80 µM	80 µM	Not specified	[4]
**Melittin**	11.4 µM	2.2 µM ATCC 27853	4.4 µM (ATCC 25923)	2.2 µM (ATCC 29212)	[5-7]
**LL-37 (17-29)**	5 µM (ATCC 25922)	116.28 µM (ATCC 19660)	40 µM (ATCC 29213)	Not specified	[8, 9]

Note: The MIC values reported for reference antimicrobial peptides were obtained from independent literature studies and are included for contextual benchmarking purposes only. Direct quantitative comparisons may be influenced by variations in experimental parameters, including bacterial strain selection, growth medium composition, inoculum size, peptide purity, counterion composition, incubation time, and antimicrobial susceptibility testing protocols.

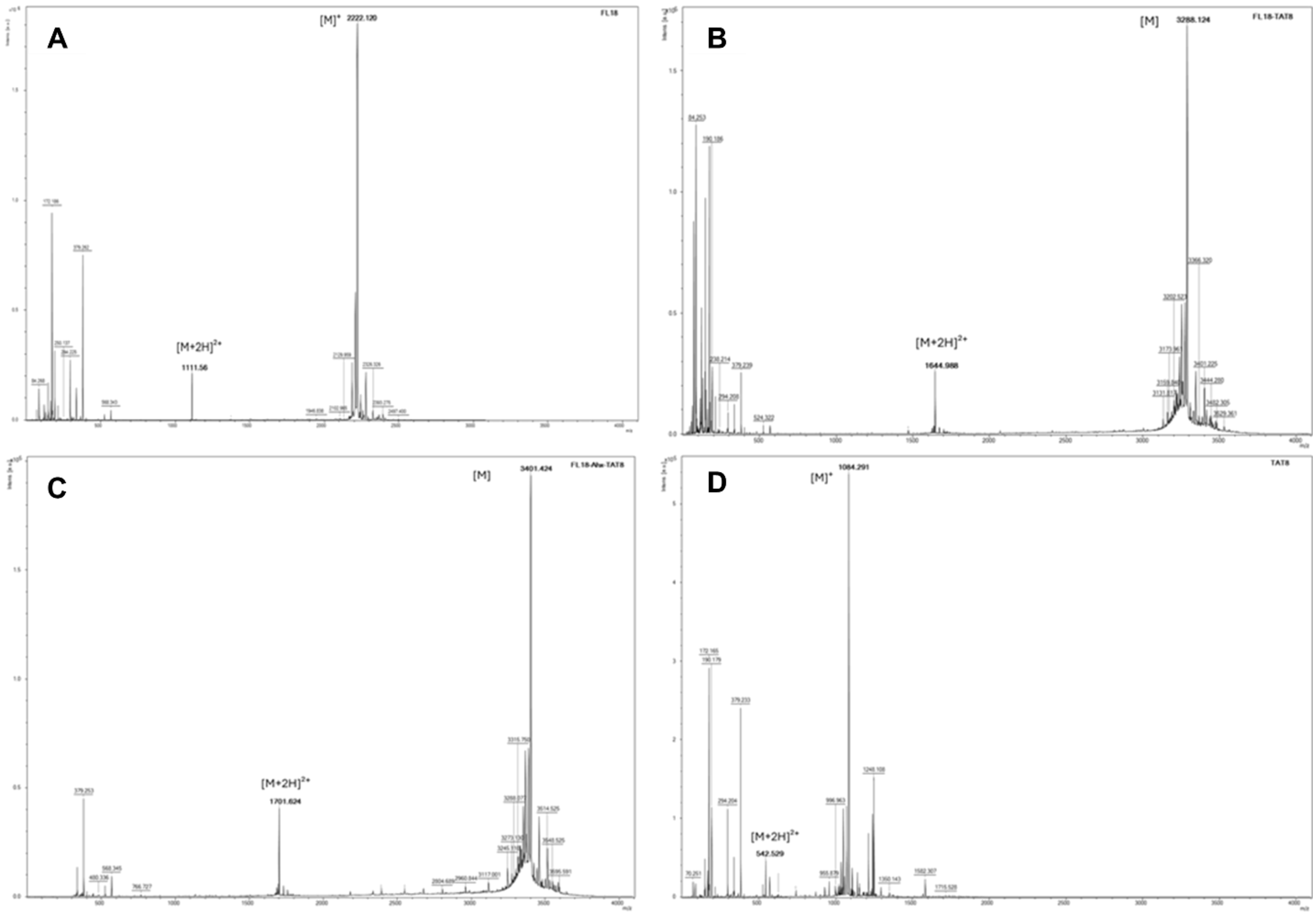

Figure S1. MALDI-TOF mass spectra of the synthesized peptides: (A) FL18, (B) FL18–TAT8, (C) FL18–Ahx-TAT8, and (D) TAT8.

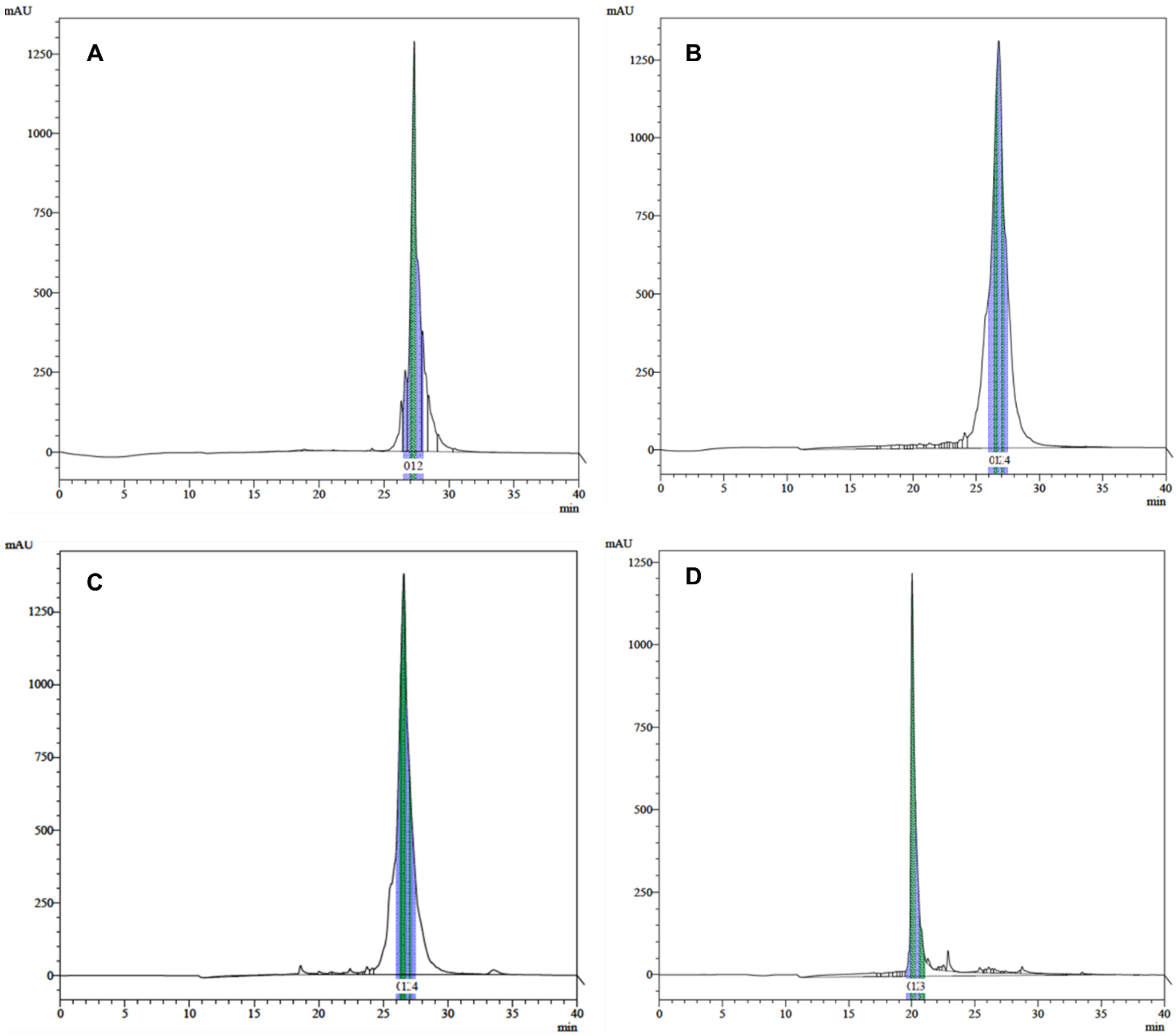

Figure S2. Prep-RP-HPLC chromatograms of the crude peptides: (A) FL18, (B) FL18–TAT8, (C) FL18–Ahx–TAT8, and (D) TAT8. The chromatograms show the major peaks corresponding to the target peptides.

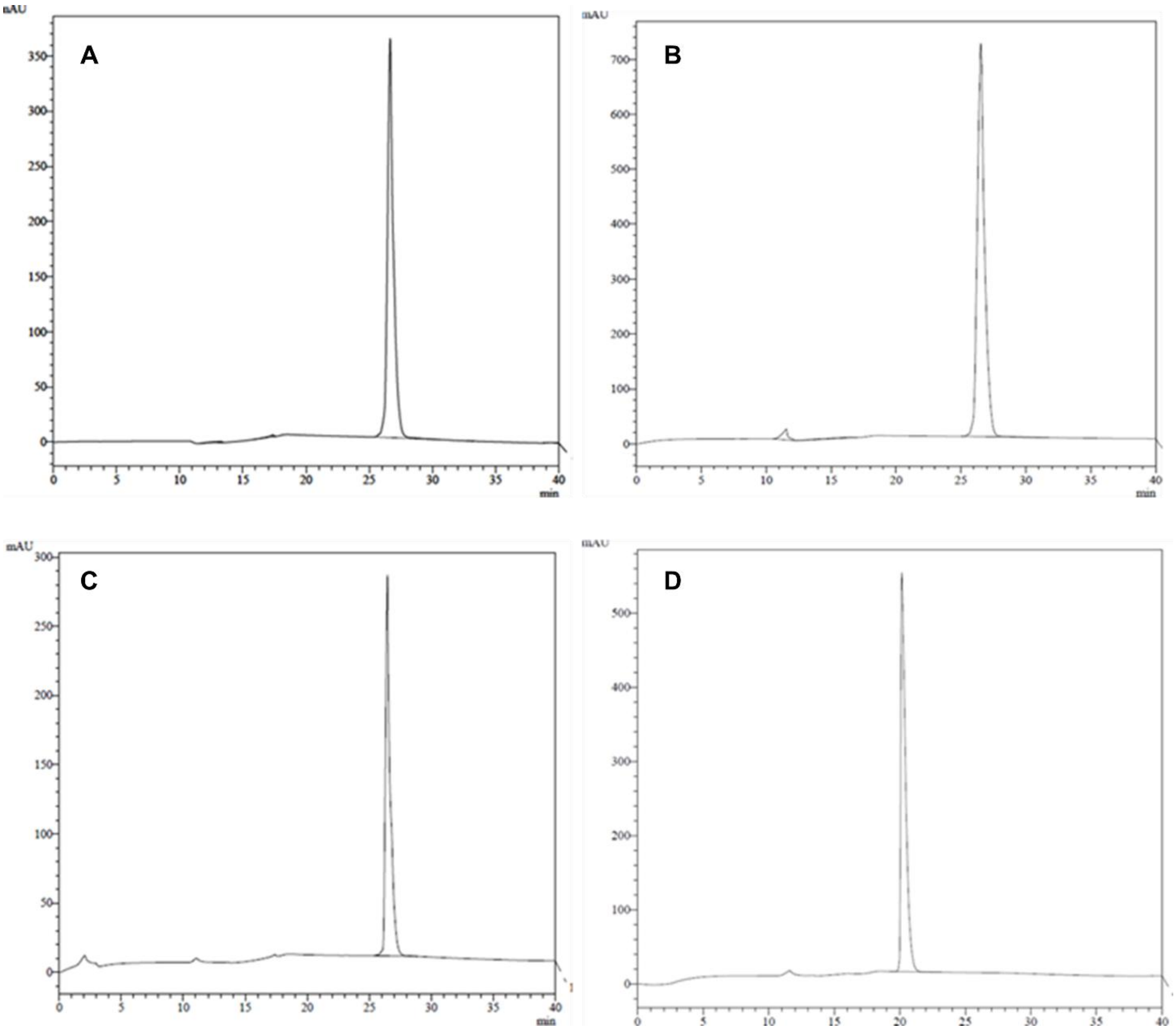

Figure S3. Analytical HPLC chromatograms of the purified peptides: (A) FL18, (B) FL18–TAT8, (C) FL18–Ahx–TAT8, and (D) TAT8. The chromatograms were recorded after purification to confirm peptide purity.

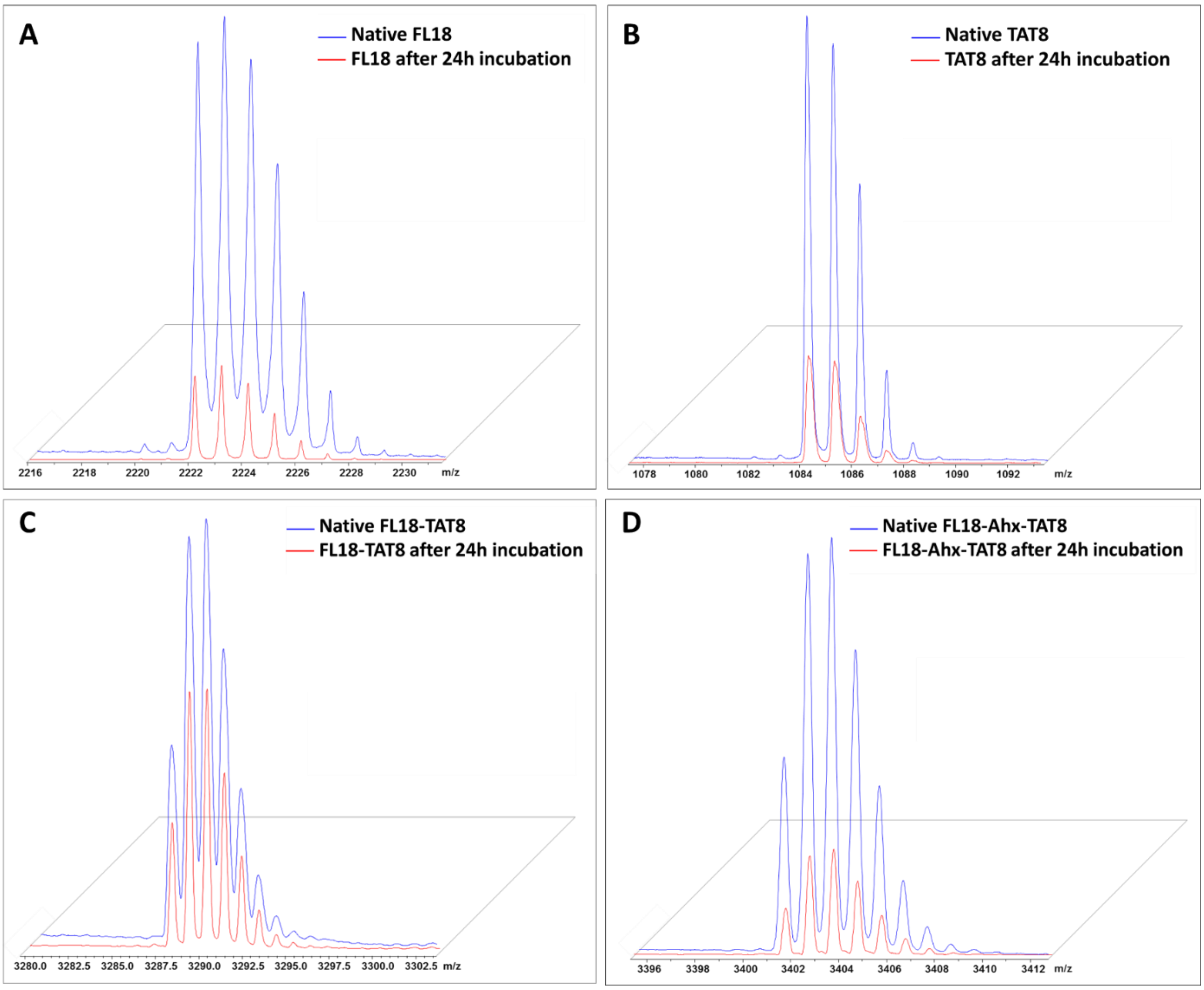

Figure S4. MALDI-TOF-MS spectra of (A) FL18, (B) TAT8, (C) FL18–TAT8, and (D) FL18–Ahx–TAT8 before and after trypsin incubation. Peptides were incubated at a final concentration of 10 µM with trypsin at a peptide-to-trypsin ratio of 1:40 in pH 7.8 buffer at 37 °C for 24 h. MALDI-TOF-MS analysis was performed using α-cyano-4-hydroxycinnamic acid (HCCA) as the matrix.

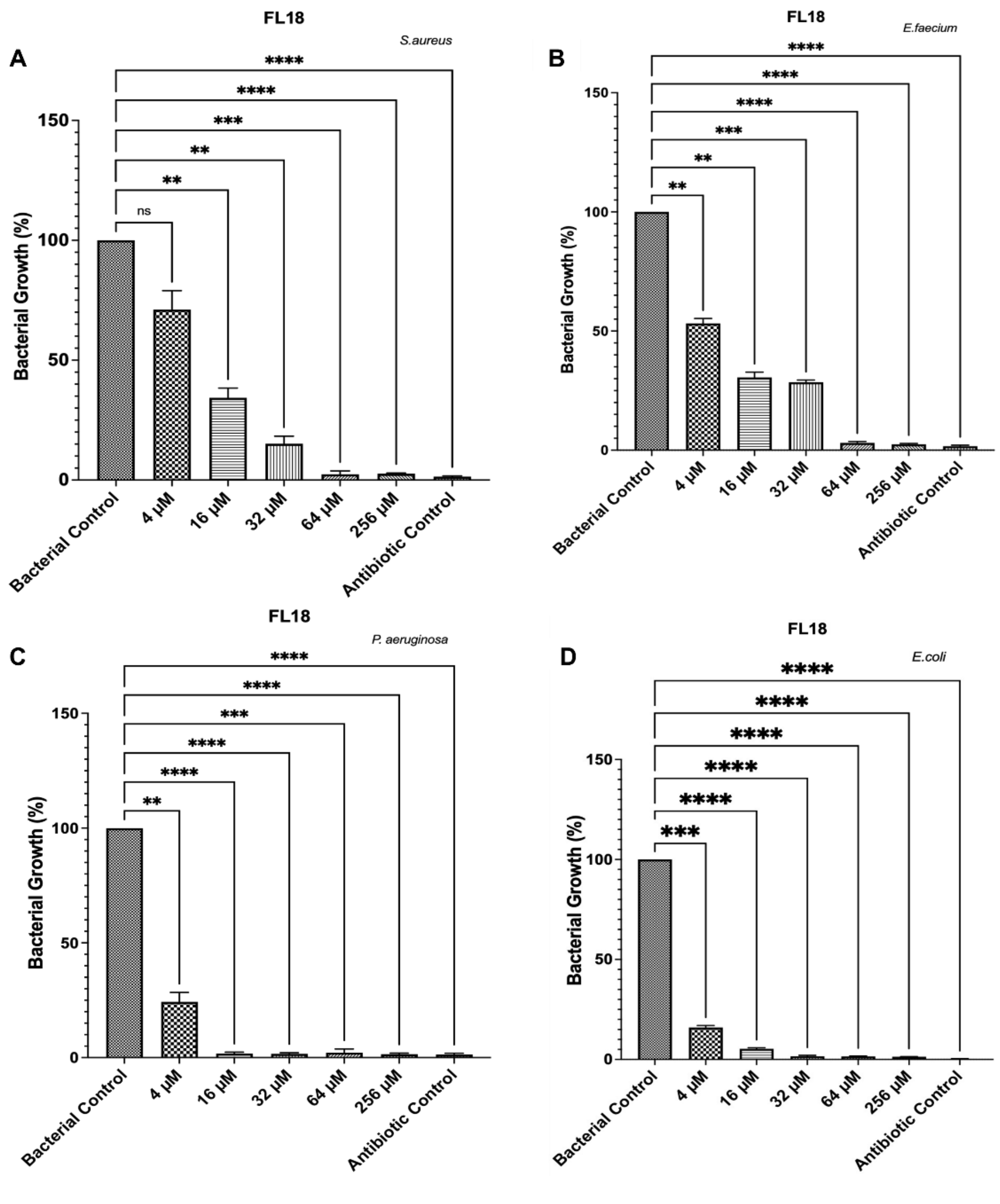

Figure S5. Antibacterial activity of FL18 against (A) *Staphylococcus aureus*, (B) *Enterococcus faecium*, (C) *Pseudomonas aeruginosa*, and (D) *Escherichia coli*, determined by the broth microdilution method. MIC values were obtained based on concentration-dependent inhibition of bacterial growth. Comparisons among multiple groups were performed using one-way ANOVA followed by Bonferroni's post hoc test. All tests were two-sided. Statistical significance was defined as follows: ns, not significant ($p > 0.05$); $*p < 0.05$; $**p < 0.01$; $***p < 0.001$; $****p < 0.0001$.

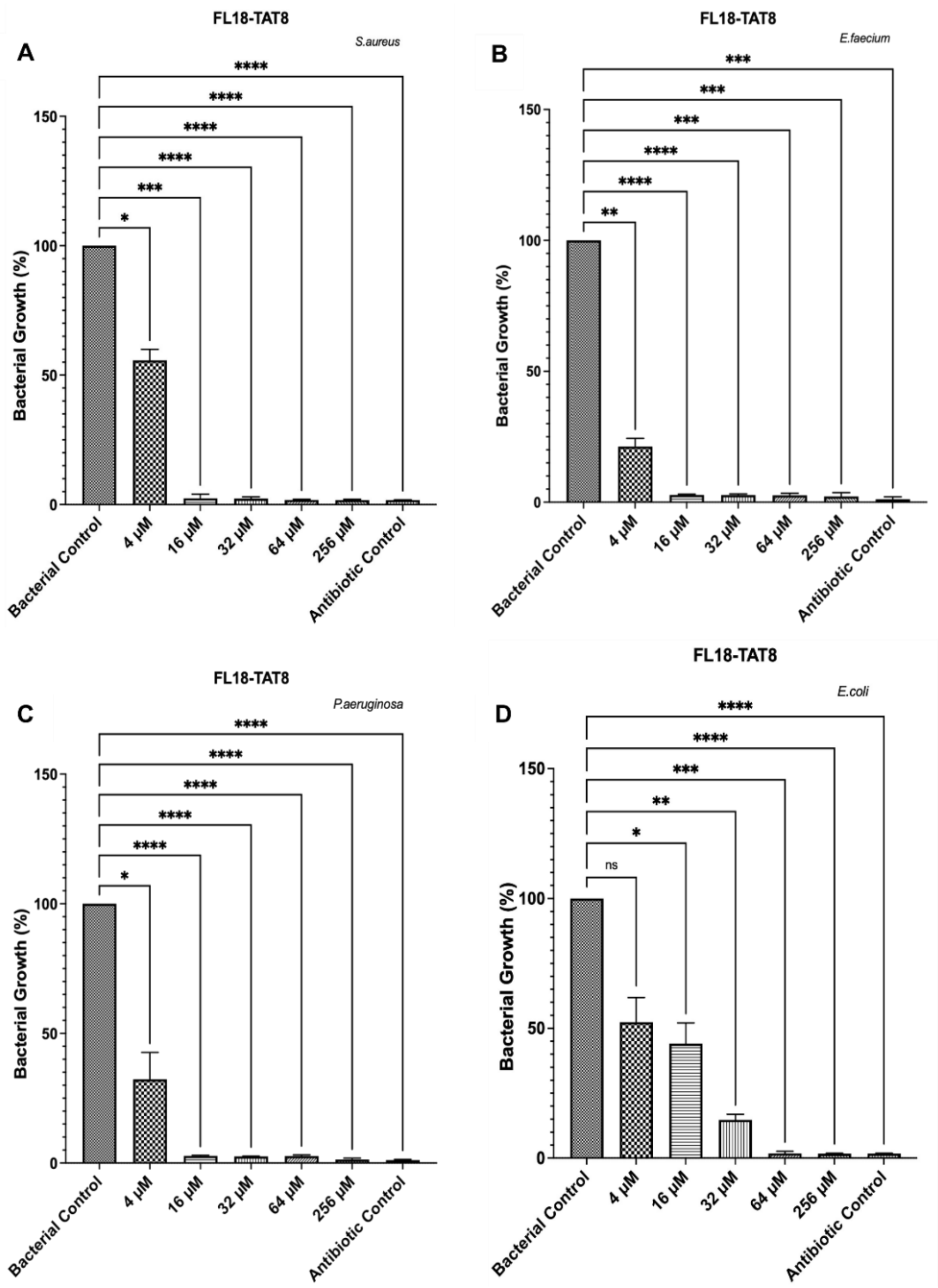

Figure S6. Antibacterial activity of FL18-TAT8 against (A) *Staphylococcus aureus* (B) *Enterococcus faecium*, (C) *Pseudomonas aeruginosa*, and (D) *Escherichia coli*, determined by the broth microdilution method. MIC values were obtained based on concentration-dependent inhibition of bacterial growth. Comparisons among multiple groups were performed using one-way ANOVA followed by Bonferroni's post hoc test. All tests were two-sided. Statistical significance was defined as follows: ns, not significant ($p > 0.05$); *$p < 0.05$; **$p < 0.01$; ***$p < 0.001$; ****$p < 0.0001$.

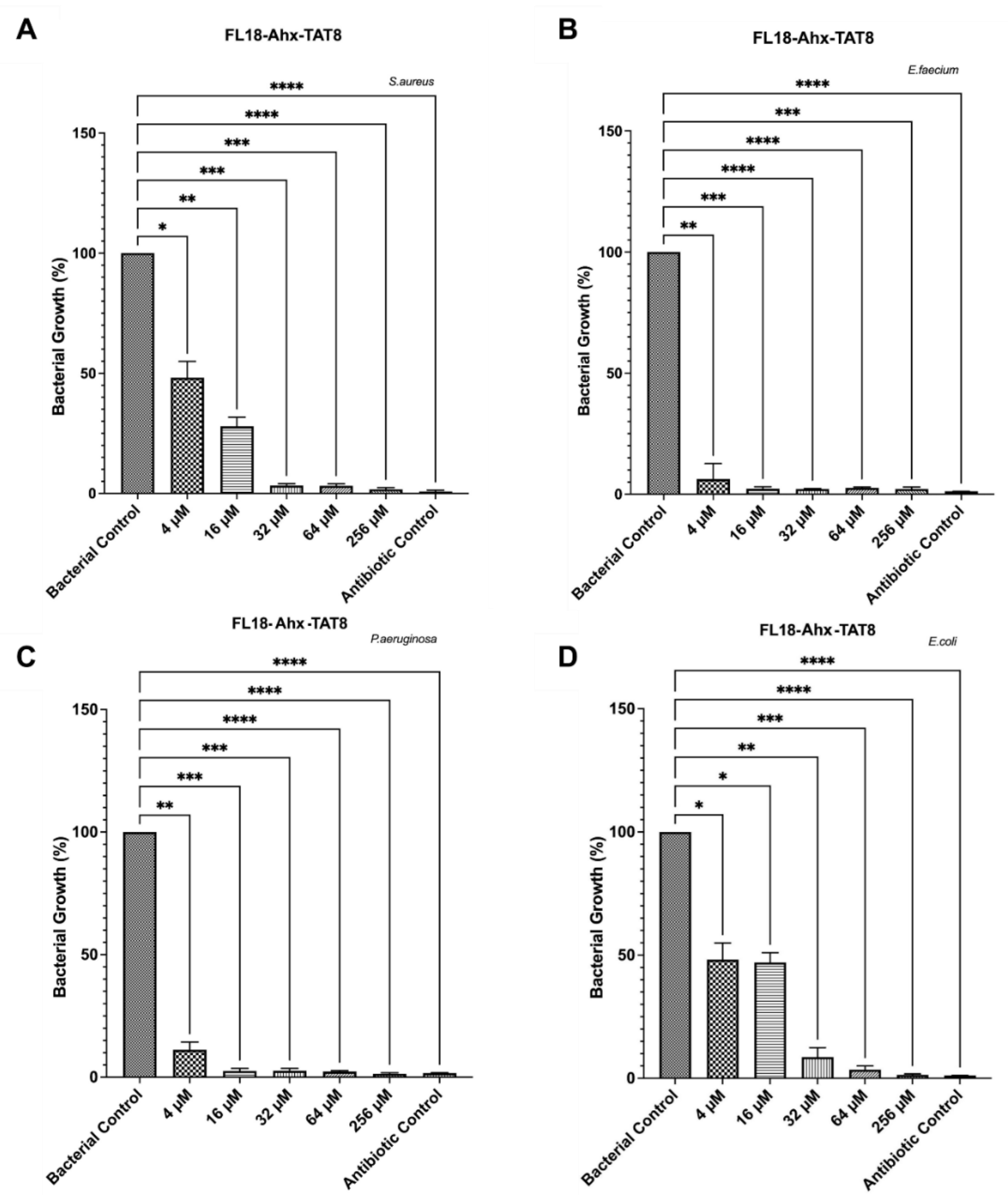

Figure S7. Antibacterial activity of FL18–Ahx–TAT8 against (A) *Staphylococcus aureus*, (B) *Enterococcus faecium*, (C) *Pseudomonas aeruginosa*, and (D) *Escherichia coli*, determined by the broth microdilution method. Comparisons among multiple groups were performed using one-way ANOVA followed by Bonferroni's post hoc test. All tests were two-sided. Statistical significance was defined as follows: ns, not significant ($p > 0.05$); *$p < 0.05$; **$p < 0.01$; ***$p < 0.001$; ****$p < 0.0001$.

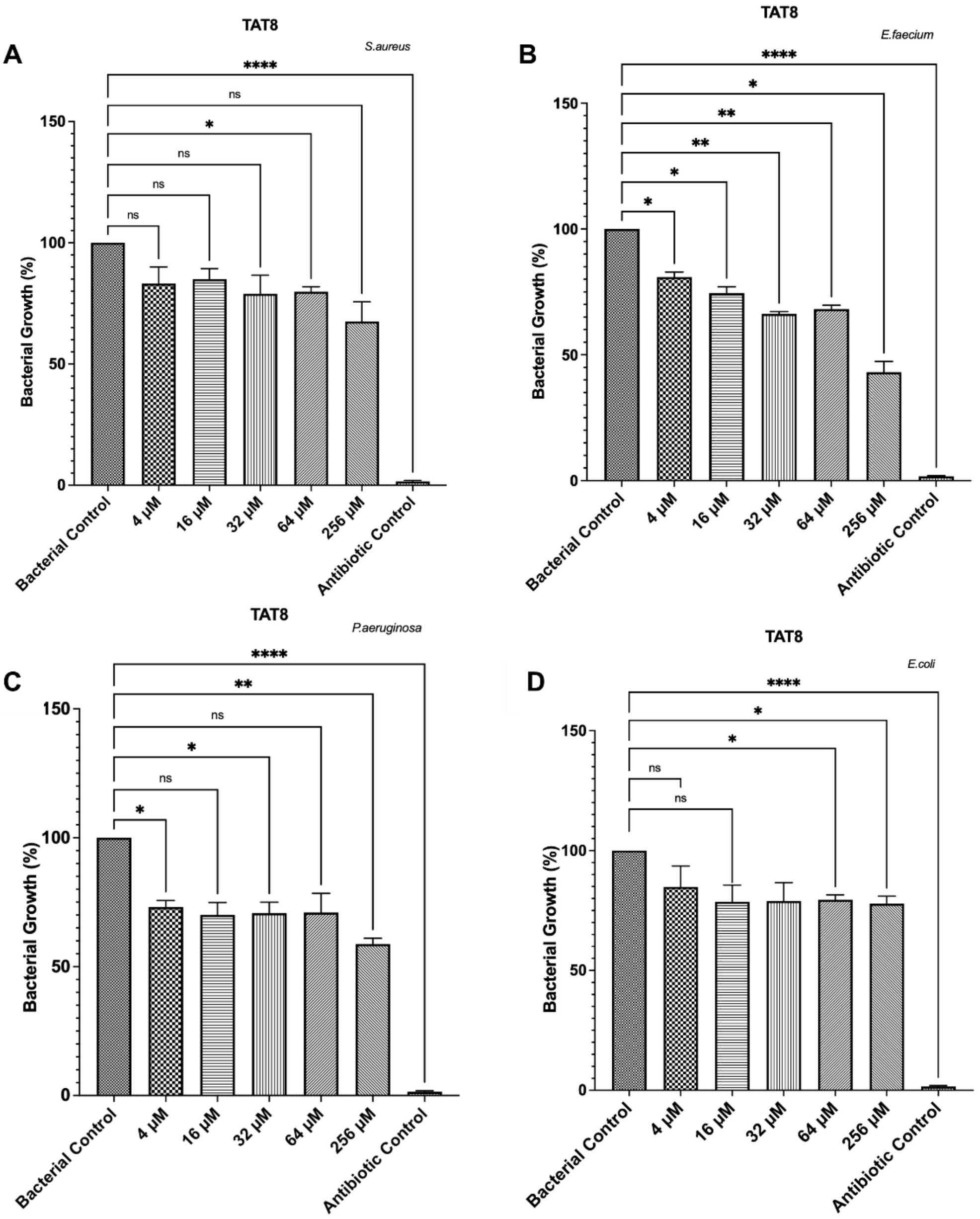

Figure S8. Antibacterial activity of TAT8 against (A) *Staphylococcus aureus*, (B) *Enterococcus faecium*, (C) *Pseudomonas aeruginosa*, and (D) *Escherichia coli*, determined by the broth microdilution method. Comparisons among multiple groups were performed using one-way ANOVA followed by Bonferroni's post hoc test. All tests were two-sided. Statistical significance was defined as follows: ns, not significant ($p > 0.05$); *$p < 0.05$; **$p < 0.01$; ***$p < 0.001$; ****$p < 0.0001$.

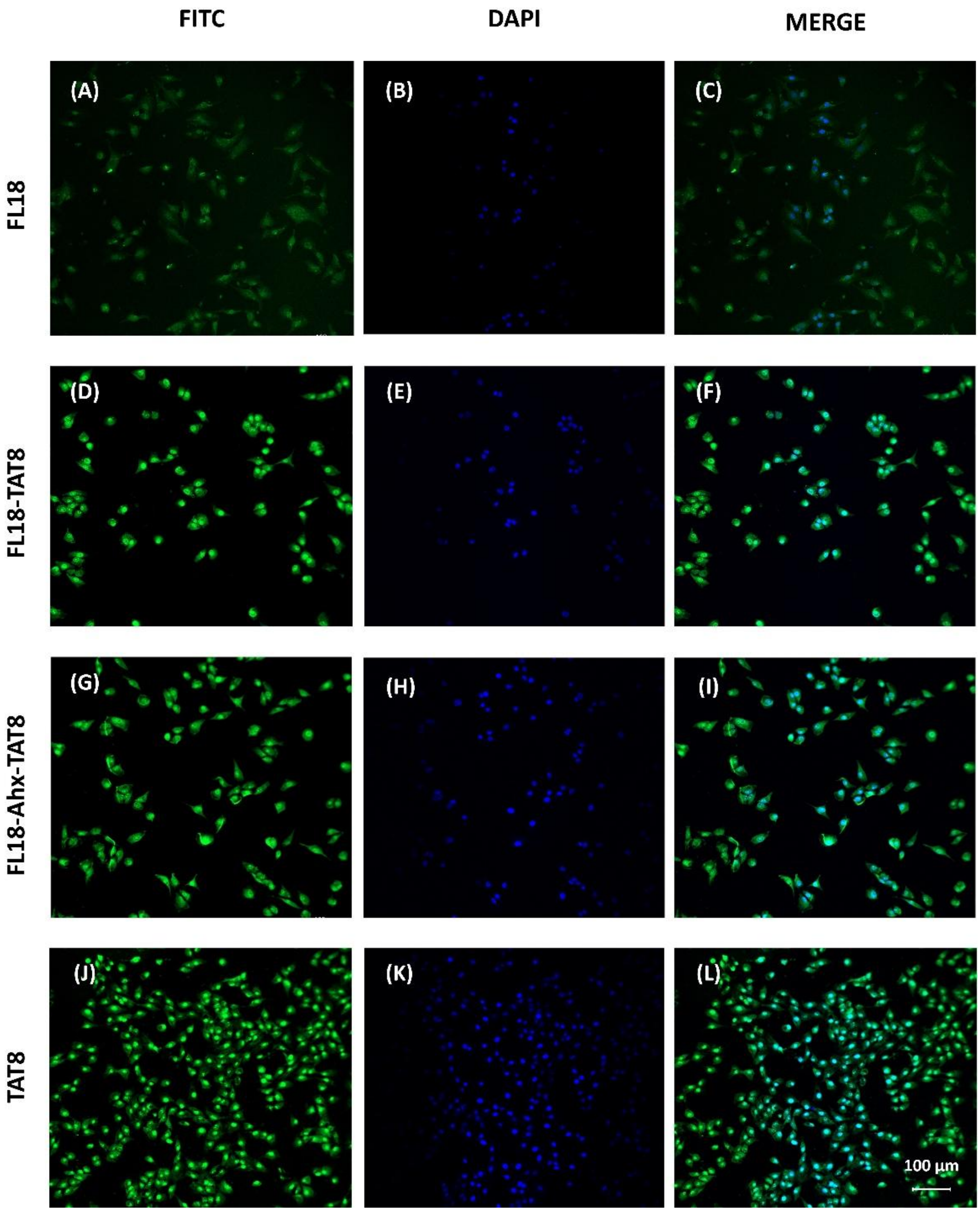

Figure S9. Confocal laser scanning microscopy images of HaCaT cells treated with FITC-labeled peptides (16 µM) for 4 h Representative images are shown for FL18 (A–C), FL18–TAT8 (D–F), FL18–Ahx–TAT8 (G–I), and TAT8 (J–L). Nuclei were stained with DAPI (blue), and peptide-associated fluorescence is shown in green. Scale bars: 100 µm.

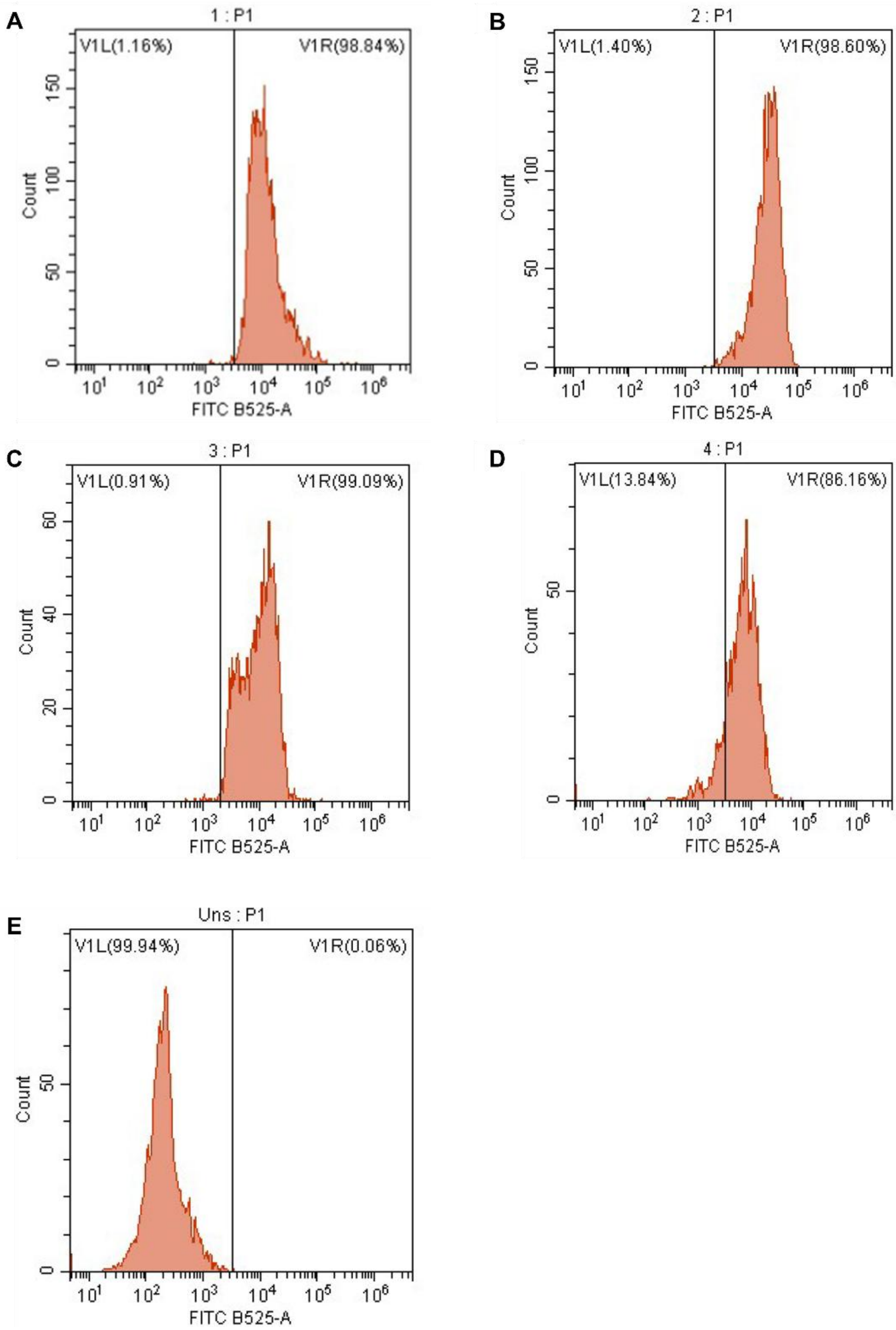

Figure S10. Fluorescence intensity of peptide-associated HaCaT cells was analyzed by flow cytometry following 1 h incubation at 25 °C in PBS (pH 7.4). Representative histograms are shown for FL18 (A), FL18–TAT8 (B), FL18–Ahx-TAT8 (C), TAT8 (D), and the untreated control (E).

References

(1) Tan, T.; Wu, D.; Li, W.; Zheng, X.; Li, W.; Shan, A. High specific selectivity and membrane-active mechanism of synthetic cationic hybrid antimicrobial peptides based on the peptide FV7. *International journal of molecular sciences* **2017**, *18* (2), 339.
(2) Yang, Y.; Wu, D.; Wang, C.; Shan, A.; Bi, C.; Li, Y.; Gan, W. Hybridization with insect cecropin A (1–8) improve the stability and selectivity of naturally occurring peptides. *International Journal of Molecular Sciences* **2020**, *21* (4), 1470.
(3) Moosazadeh Moghaddam, M.; Eftekhary, M.; Erfanimanesh, S.; Hashemi, A.; Fallah Omrani, V.; Farhadihosseinabadi, B.; Lasjerdi, Z.; Mossahebi-Mohammadi, M.; Pal Singh Chauhan, N.; Seifalian, A. M. Comparison of the antibacterial effects of a short cationic peptide and 1% silver bioactive glass against extensively drug-resistant bacteria, Pseudomonas aeruginosa and Acinetobacter baumannii, isolated from burn patients. *Amino acids* **2018**, *50* (11), 1617-1628.
(4) Dathe, M.; Wieprecht, T.; Nikolenko, H.; Handel, L.; Maloy, W. L.; MacDonald, D. L.; Beyermann, M.; Bienert, M. Hydrophobicity, hydrophobic moment and angle subtended by charged residues modulate antibacterial and haemolytic activity of amphipathic helical peptides. *FEBS letters* **1997**, *403* (2), 208-212.
(5) Yan, H.; Li, S.; Sun, X.; Mi, H.; He, B. Individual substitution analogs of Mel (12–26), melittin's C-terminal 15-residue peptide: their antimicrobial and hemolytic actions. *FEBS letters* **2003**, *554* (1-2), 100-104.
(6) Sung, W. S.; Park, S. H.; Lee, D. G. Antimicrobial effect and membrane-active mechanism of Urechistachykinins, neuropeptides derived from Urechis unicinctus. *FEBS letters* **2008**, *582* (16), 2463-2466.
(7) Sun, X.; Chen, S.; Li, S.; Yan, H.; Fan, Y.; Mi, H. Deletion of two C-terminal Gln residues of 12–26-residue fragment of melittin improves its antimicrobial activity. *Peptides* **2005**, *26* (3), 369-375.
(8) Son, M.; Lee, Y.; Hwang, H.; Hyun, S.; Yu, J. Disruption of interactions between hydrophobic residues on nonpolar faces is a key determinant in decreasing hemolysis and increasing antimicrobial activities of α-helical amphipathic peptides. *ChemMedChem* **2013**, *8* (10), 1638-1642.
(9) Mohammed, I.; Said, D. G.; Nubile, M.; Mastropasqua, L.; Dua, H. S. Cathelicidin-derived synthetic peptide improves therapeutic potential of vancomycin against Pseudomonas aeruginosa. *Frontiers in microbiology* **2019**, *10*, 2190.
